# CRB-701: A Second-generation Nectin-4–Targeted Antibody–Drug Conjugate with Optimized Stability and Pharmacokinetics for the Treatment of Solid Tumors

**DOI:** 10.1158/2767-9764.CRC-25-0799

**Published:** 2026-09-15

**Authors:** Zhaopeng Sun, Mo Dan, Lu Lv, Mingyue Shen, Can Yuan, Congcong Niu, Yang Zhang, Xixin Hu, Xiwu Hui, Andrew Kolodziej

**Affiliations:** 1 CSPC Megalith Biopharmaceutical Co., Ltd., Shijiazhuang, China.; 2 Corbus Pharmaceuticals Inc., Norwood, Massachusetts.

## Abstract

The adhesion protein nectin-4 is a clinically validated tumor-selective antigen that is overexpressed across multiple cancer types and has been successfully exploited as a target for antibody–drug conjugates (ADCs), most notably enfortumab vedotin (EV), which is approved for the treatment of urothelial carcinoma. In this study, we report the design and preclinical characterization of a second-generation nectin-4–targeted ADC, CRB-701 (also identified as SYS6002). CRB-701 was synthesized using microbial transglutaminase technology to conjugate two molecules of monomethyl auristatin E (MMAE) via a cleavable linker to a highly selective, high-affinity anti–nectin-4 monoclonal antibody. CRB-701 exhibited potent *in vitro* cytotoxicity in nectin-4–expressing cell lines and *in vivo* antitumor activity in cell line-derived and patient-derived murine xenograft models of human cancer (cell line-derived models: PC-3-NECTIN4 prostate cancer, MDA-MB-468 breast cancer, and HT1376 bladder cancer; patient-derived model: BL0597 bladder cancer), exhibiting efficacy similar to or greater than EV across tumors with varying levels of nectin-4 expression. CRB-701 delivered high levels of MMAE upon internalization in tumor cells and was markedly more stable in circulation than EV, with lower systemic MMAE release, an approximately twofold longer half-life, and at least a twofold higher safety margin in monkeys. These results suggest that CRB-701 may be a promising alternative therapeutic for the treatment of nectin-4–expressing tumors, providing a more favorable therapeutic window and potentially enabling regimens with higher doses and less frequent dosing than those required for EV, the only currently approved nectin-4–targeting ADC.

**Significance:**

CRB-701 is a nectin-4–targeted ADC. CRB-701 showed similar or superior antitumor activity, better stability in plasma, and was associated with lower systemic MMAE release than EV, the only approved nectin-4–targeted ADC. CRB-701 offers a promising alternative for treatment of nectin-4–expressing tumors.

## Introduction

Antibody–drug conjugates (ADCs) have emerged as a leading therapeutic modality in oncology owing to their high tumor specificity and unprecedented response rates in cancer types that are otherwise refractory to treatment (1, 2). ADCs typically comprise a potent small-molecule cytotoxic agent that is conjugated to a tumor-selective antibody via a cleavable linker. Following receptor-mediated internalization by the tumor cell, the ADC undergoes lysosomal processing and the cytotoxic payload is released through linker cleavage, ultimately inducing cell death (3). ADCs may also be engineered to induce immune effector action, for example, complement-dependent cytotoxicity (CDC) and antibody-dependent cellular cytotoxicity (ADCC), which can further boost antitumor activity (4).

This strategy of leveraging antibodies to deliver cytotoxic agents directly to tumor cells aims to provide a favorable therapeutic index while mitigating the dose-limiting toxicities typically associated with systemic delivery of cytotoxic agents (2). Nevertheless, ADCs are also associated with significant toxicities, partly due to uptake in nontumor tissues in which the target antigen may be expressed at low levels and partly due to systemic release of the cytotoxic payload through nonspecific linker hydrolysis (5, 6). To address these challenges, considerable efforts have been directed toward tuning the drug:antibody ratio (DAR), optimizing linker stability, and refining antibody–drug conjugation methods to minimize off-target payload release (7, 8).

Nectin-4 (encoded by the *PVRL4* gene) has emerged as an important tumor-selective antigen because of its low expression in healthy adult tissues and its upregulation across a range of tumor types, including urothelial (9), breast (10), lung (11), pancreatic (12), ovarian (13), head/neck (14), and esophageal cancers (15). Moreover, nectin-4 is an independent biomarker for poor overall survival in numerous cancer types (16) and is therefore well established as a therapeutic target. Despite this, only one nectin-4–targeted therapy, enfortumab vedotin (EV), has received regulatory approval to date (17).

EV is a monomethyl auristatin E (MMAE)-based nectin-4–targeted ADC. It is approved in combination with the anti-programmed cell death protein 1 (PD-1) agent pembrolizumab as a first-line treatment for patients with locally advanced or metastatic urothelial cancer and as a monotherapy for patients who have previously received a PD-1 or programmed cell death ligand 1 inhibitor and platinum-containing chemotherapy (17). However, its use has not yet been approved in other tumor types. EV is associated with a high incidence of toxicities, many of which can be dose limiting, including fatigue (51% of patients), ocular disorders (40% of patients), skin reactions (58% of patients), and peripheral neuropathy (53% of patients; ref. 17). In clinical trials, adverse reactions led to dose interruptions in 64% of patients and dose reductions in 34% of patients (17), limiting the duration and intensity of treatment and potentially compromising long-term efficacy. Finally, treatment with EV monotherapy requires weekly intravenous infusions (17), which may be burdensome for the patient and may negatively affect treatment compliance.

EV employs maleimide chemistry to attach the linker payload to the antibody, yielding a DAR of approximately 3.8 (17). However, the antibody–linker thioether bond is susceptible to reverse Michael addition, which may contribute to premature systemic release of the cytotoxic payload (18). MMAE exposure increases with EV dose, potentially giving rise to toxicities and the need for frequent dose modifications (19). There is, therefore, growing interest in the development of novel nectin-4–targeted therapies offering enhanced intratumoral delivery of cytotoxic agents, with more favorable safety profiles and dosing regimens.

Here, we report the design and preclinical characterization of a novel nectin-4–targeted ADC, CRB-701. CRB-701 comprises an immunoglobulin G1 (IgG1) humanized nectin-4 antibody that is conjugated to MMAE using microbial transglutaminase (mTGase) technology. This method forms stable isopeptide bonds between the antibody and MMAE–linker payload (20), aiming to provide greater conjugation stability than the maleimide chemistry employed by EV. Here, we demonstrate that CRB-701 exhibits specific and selective binding to nectin-4 and potent antitumor activity in multiple murine xenograft models for human cancer. These factors, coupled with a favorable pharmacokinetic (PK) and safety profile, suggest that CRB-701 may have promising therapeutic potential in a range of nectin-4–expressing tumors.

## Materials and Methods

Details of key materials used in all assays are available in Supplementary Methods and Supplementary Table S1.

### Cell lines

PC-3 parental cells (CoBioer; RRID: CVCL_0035) and a PC-3 cell line stably expressing nectin-4 (PC-3-NECTIN4; Kyinno Biotechnology) were cultured in Dulbecco’s Modified Eagle Medium (DMEM)/F12 complete medium (Gibco) supplemented with 10% fetal bovine serum (FBS; Gibco) and 1 μg/mL puromycin (Sigma/Gibco). The parental renal cell line 293T (Kyinno Biotechnology; RRID: CVCL_0063) and a 293T cell line stably expressing nectin-4 (293T-NECTIN4; Kyinno Biotechnology; RRID: CVCL_E8M6) were cultured in DMEM supplemented with 10% FBS and 1 μg/mL puromycin. HEK293 cells stably expressing luciferase (HEK293-LUC; Shanghai Meixuan Biological; RRID: CVCL_C9C7) were cultured in DMEM (HyClone) containing 10% FBS. Breast cancer cell lines T47D (RRID: CVCL_0553), SK-BR-3 (RRID: CVCL_0033), and MDA-MB-468 (RRID: CVCL_0419) were obtained from the American Type Culture Collection and cultured in DMEM supplemented with 10% FBS. Jurkat/hFcγRIIIa-NFAT cells were obtained from the National Institutes for Food and Drug Control and cultured in 88% Roswell Park Memorial Institute basal medium (HyClone) supplemented with 10% FBS, 1 mmol/L sodium pyruvate, 500 μg/mL G418, and 1 μg/mL puromycin. All cells were maintained in T75 culture flasks at 37°C in a humidified incubator with 5% CO_2_ and were minimally passaged after thawing. All cell lines were confirmed to be mycoplasma free prior to use, using standard testing in the US Consumer Product Safety Commission laboratories, and were validated by short tandem repeat profiling.

### CRB-701 parent antibody and ADC generation

The parent CRB-701 monoclonal antibody (CRB-701-mAb) and the MMAE-conjugated ADC CRB-701 were manufactured by CSPC Megalith Biopharmaceutical Co., Ltd. CRB-701-mAb is an IgG1 derived from BALB/c mice (RRID: MGI:2161072) using hybridoma technology that was further optimized by sequence humanization. The heavy and light chains were stably coexpressed in a Chinese hamster ovary cell line (CHOK1SV GS-KO; Lonza; RRID: CVCL_DR97) and purified using affinity and ion exchange chromatography. CRB-701-mAb was conjugated to two molecules of LND1002 at the Q294 residue of each heavy chain via an amide bond, catalyzed by mTGase (20). LND1002 is a linker composed of three functional moieties: an amino polyethylene glycol (PEG) spacer, a valine-citrulline *p*-amino benzyloxycarbonyl cathepsin B cleavable site (21), and the cytotoxic payload MMAE.

### Nectin-4 and Fc binding affinity

Sandwich enzyme-linked immunosorbent assays (ELISA) were performed to assess the binding of CRB-701 to nectin-4. Briefly, 96-well polystyrene plates were coated overnight at 4°C with recombinant human, mouse, rat, or cynomolgus monkey nectin-4 (0.5 μg/mL in carbonate coating buffer). Plates were blocked with 1% bovine serum albumin in phosphate-buffered saline (PBS) for 2 hours at room temperature and then incubated with sixfold serial dilutions of CRB-701 (starting at 5 μg/mL). After washing, bound antibody was detected with horseradish peroxidase–conjugated goat anti-human IgG F(ab’)_2_ (Jackson ImmunoResearch; RRID: AB_2337596) and 3,3′,5,5′‑tetramethylbenzidine substrate. Absorbance (450/650 nm) was read using a microplate reader, and the half-maximal effective concentration (EC_50_) was calculated by fitting the dose–response data to a four-parameter logistic model.

Binding affinity of CRB-701/CRB-701-mAb to human nectin-4 protein, human Fc receptors, and human C1q protein was assessed via biolayer interferometry (BLI) using an Octet RED96e System (Pall FortéBio). Recombinant human nectin-4 protein was immobilized onto HIS1K biosensor chips, and binding responses were measured following exposure to vehicle, CRB-701, or CRB-701-mAb starting at initial concentrations of 200 nmol/L (approximately 30 μg/mL), followed by seven twofold serial dilutions down to 1.6 nmol/L. Experimental conditions for human Fc receptors and human C1q protein BLI assays are shown in Supplementary Methods. Association rate constants (k_on_) and dissociation rate constants (k_off_) were determined by fitting the response-versus-time data to a global 1:1 Langmuir binding model using the instrument’s software. The binding affinity (K_d_) was calculated from these kinetic parameters (K_d_ = k_off_/k_on_).

### Binding of CRB-701 and CRB-701-mAb to nectin-4–expressing cells

Binding of CRB-701 to nectin-4–expressing cells was assessed by flow cytometry and used as a proxy for relative nectin-4 expression in PC-3-NECTIN4, 293T-NECTIN4, SK-BR-3, and MDA-MB-468 cells. Cells (1 × 10^6^ cells/mL, 100 μL per well) were seeded into 96-well plates. Following treatment with 5 μg/mL CRB-701 or CRB-701-mAb, cells were incubated for 60 to 90 minutes at 4°C, pelleted by centrifugation, washed, and labeled with Alexa Fluor 488-conjugated goat anti-human IgG (H + L; Invitrogen; RRID: AB_2534080) cross-adsorbed secondary antibody (1:1,000 dilution). Cells were then collected by centrifugation (1,000 × *g*, 5 minutes). The median fluorescence intensity (MFI) of bound antibody was quantified using an Attune NxT flow cytometer (Thermo Fisher Scientific; RRID: SCR_019590).

### Cytotoxicity assays

Cytotoxicity assays were performed using PC-3, PC-3-NECTIN4, 293T, 293T-NECTIN4, SK-BR-3, and MDA-MB-468 cell lines. Cells were seeded in 96-well plates at a density of 1 × 10^5^ cells/mL (100 μL per well) and treated with 10 or 11 twofold (SK-BR-3 cells) or threefold (PC-3, PC-3-NECTIN4, 293T, 293T-NECTIN4, and MDA-MB-468 cells) serial dilutions of EV, CRB-701, or CRB-701-mAb, to obtain maximum final concentrations of 0.833 μg/mL (293T and 293T-NECTIN4 cells), 1.667 μg/mL (PC-3 and PC-3-NECTIN4 cells), 500 μg/mL (MDA-MB-468 cells), or 1000 μg/mL (SK-BR-3).

Cells were then incubated for 63 to 69 hours at 37°C and 5% CO_2_. Resazurin solution (0.03%) was added to the plate at 20 μL/well, and the plate was incubated at 37°C for a further 3 to 4 hours. Fluorescence values were read at 550 nm/610 nm using a microplate reader with the relative fluorescence unit (RFU) used as an indicator of cell viability. Dose–response curves were generated by fitting RFU versus concentration data to a four-parameter logistic model using nonlinear regression (Prism version 10.1.1, GraphPad; RRID: SCR_002798), and IC_50_ values (the concentration required to achieve 50% maximal inhibition) were calculated.

To evaluate bystander cytotoxicity, nectin-4–expressing cells (293T-NECTIN4 or PC-3-NECTIN4) were cocultured with HEK293-LUC cells, which stably express luciferase but lack nectin-4 expression. Cells were cultured to 90% confluence, detached using trypsin, and suspended at a density of 2 × 10^5^ cells/mL. Nectin-4–expressing cells were mixed 1:1 with HEK293-LUC cells and seeded into 96-well plates (100 μL per well). For the 293T-NECTIN4 coculture, CRB-701-mAb or CRB-701 was added to obtain final concentrations of 16.7, 3.33, 0.67, 0.13, and 0.027 μg/mL. For the PC-3-NECTIN4 coculture, CRB-701-mAb or CRB-701 was added to obtain concentrations of 8.33, 1.67, 0.67, 0.17, 0.042, and 0.0083 μg/mL. After 72 hours of incubation, luminescence was measured using a microplate luminometer following the addition of Bright-Glo Luciferase Reagent (Promega), providing a quantitative readout of HEK293-LUC cell viability.

### Endocytosis and MMAE release

CRB-701 endocytosis was investigated in PC-3-NECTIN4 cells, and MMAE release was investigated in PC-3-NECTIN4 and the parent cells (PC-3), which do not express nectin-4 (22). For the endocytosis assay, PC-3-NECTIN4 cells were seeded at a density of 2.5 × 10^5^ cells/mL in 6-well plates and incubated overnight at 37°C. The following day, cells were treated with 2 μg/mL of either CRB-701 or CRB-701-mAb and incubated at 4°C for 2 hours to allow surface binding. Excess antibody was removed by washing with Dulbecco’s PBS (DPBS), and cells were then incubated at 37°C and 5% CO_2_ for 0, 3, 6, 9, 24, or 48 hours to initiate internalization. Cells were then transferred to a 96-well plate, and surface-bound antibodies were detected using flow cytometry with Alexa Fluor 488-conjugated goat anti-human IgG (H + L) cross-adsorbed secondary antibody (Invitrogen). Endocytosis percentages were calculated based on MFI using the formula: [Endocytosis % = (MFI_t0_ – MFI_tx_)/MFI_t0_ × 100], where MFI_t0_ and MFI_tx_ represent fluorescence intensity at time zero and time point x, respectively. To assess endocytosis in T47D and SK-BR-3 cells, 1 × 10^4^ cells/well were added to 96-well plates and incubated with 2 μg/mL of CRB-701 or EV and fluorescent Zenon pHrodo iFL IgG Labeling Reagents (Invitrogen) for up to 24 hours at 37°C and 5% CO_2_. Internalization was measured using a laser confocal microscope equipped with a 20× objective lens.

For the intracellular MMAE release assay, PC-3-NECTIN4 and parental PC-3 cells were cultured to approximately 90% confluence and then detached using trypsin and resuspended in their respective complete media. Cells were adjusted to a density of 2.5 × 10^5^ cells/mL, seeded into 6-well plates, and incubated overnight at 37°C. The following day, cells were treated with CRB-701 at a concentration of 2 μg/mL for 2 hours at 4°C to allow surface binding. After washing with DPBS to remove unbound antibody, cells were incubated in complete medium at 37°C and 5% CO_2_ for 6 or 24 hours to permit internalization and intracellular processing.

Following incubation, cells were collected, washed with DPBS, and lysed in RIPA buffer (Thermo Fisher Scientific). Lysates were stored at −80°C until analysis. Intracellular MMAE concentrations were quantified using liquid chromatography/tandem mass spectrometry (LC/MS-MS). The assay was performed on an AB SCIEX LC-30 system (SCIEX) equipped with an ACQUITY Ultra-Performance Liquid Chromatography ethylene bridged hybrid C18 column (1.7 μm, 2.1 × 50 mm) and an AB SCIEX Triple Quad 5500 mass spectrometer. MMAE levels were quantified against an 8-point calibration curve ranging from 0.023 to 50 ng/mL.

### Cell-cycle arrest and apoptosis assays

Cell-cycle distribution changes were assessed in 293T-NECTIN4 cells using propidium iodide (PI) staining followed by flow cytometry. Cells in the logarithmic growth phase were trypsinized, seeded at a density of 2 × 10^5^ cells/mL in 6-well plates, and preincubated for 4 to 6 hours at 37°C and 5% CO_2_. Cells were then treated with 10 μg/mL CRB-701-mAb or CRB-701, or control medium for 66 ± 3 hours. After treatment, cells were harvested, washed with DPBS twice, and then fixed in cold 70% ethanol at −20°C for 2 hours. Fixed cells were washed again and stained with a solution of RNase A and PI, followed by incubation in the dark for 30 to 60 minutes to allow DNA labeling. DNA content was quantified by flow cytometry using 488 nm excitation, and PI red fluorescence was analyzed to determine cell-cycle phase distribution.

For apoptosis assays in 293T-NECTIN4 cells, 2 × 10^5^ cells/mL in the logarithmic growth phase were treated in culture media with 10 μg/mL CRB-701 or CRB-701-mAb, or vehicle for 66 ± 3 hours at 37°C. After trypsin digestion and collection, cells were stained using the Annexin V-FITC/PI Staining Apoptosis Detection Kit (KeyGEN BioTECH) and apoptosis stage was quantified via flow cytometry.

To evaluate the activation of apoptosis in PC-3-NECTIN4 cells, 10^4^ cells/well were treated for 24 hours with 11 fourfold dilutions of CRB-701, CRB-701-mAb, or vehicle (starting concentration 100 μg/mL). Apoptosis was determined using the Caspase-Glo 3/7 Assay System (Promega) in accordance with the manufacturer’s instructions. Luminescence was measured using a microplate reader. EC_50_ values were obtained by fitting data to a four-parameter sigmoid function by nonlinear regression (GraphPad Prism version 10.1.1).

### Effector activity: CDC and ADCC

CDC activity was assessed using the CellTiter-Glo Luminescent Cell Viability Assay (Promega). Target 293T-NECTIN4 cells were seeded into 96-well plates at a density of 2 × 10^5^ cells/well and incubated with a uniform pool of human serum–derived complement (QuidelOrtho) in the presence of 10 threefold serial dilutions of CRB-701 or CRB-701-mAb (starting concentration 10 μg/mL). After 22 to 24 hours at 37°C and 5% CO_2_, 100 μL/well of luciferase assay substrate solution was added and cells were incubated in the dark for 5 to 15 minutes. Luminescence was measured as a proxy for viable cells using a microplate reader.

ADCC activity was measured using Jurkat/hFcγRIIIa-NFAT transgenic effector cells, which express luciferase under the control of an NFAT response element. Effector cells (3 × 10^6^ cells/well) were cocultured with 293T-NECTIN4 target cells (1 × 10^5^ cells/well) at a 15:1 effector:target ratio in a total volume of 75 μL. Cells were treated with fourfold serial dilutions of CRB-701 or CRB-701-mAb (final concentrations ranging from 15 μg/mL to 5.7 × 10^−5^ μg/mL) and incubated for 20 to 22 hours at 37°C and 5% CO_2_. Luciferase substrate solution was added (100 μL/well), and cells were incubated in the dark for 5 to 15 minutes. ADCC activity was quantified by measuring luciferase expression, with relative light unit (RLU) serving as an indicator of cytotoxic response.

For CDC and ADCC, dose–response data (RLU vs. concentration) were fitted to a four-parameter sigmoidal model to determine EC_50_ values.

### *In vivo* xenograft studies

All animal studies were conducted in accordance with institutional guidelines and approved protocols. Cell line-derived xenograft (CDX) studies were approved by CSPC’s Institutional Animal Care and Use Committee, and patient-derived xenograft (PDX) studies were approved by Crown Bioscience Inc.

CDX models were established in NU/NU (RRID: IMSR_CRL:088) and nonobese diabetic/severe combined immunodeficiency (NOD/SCID; RRID: IMSR_CRL:394) mice obtained from Vital River Laboratory Animal Technology Co., Ltd. PDX studies were conducted in female BALB/c nude mice (RRID: IMSR_CRL:194) supplied by Beijing Anikeeper Biotech Co., Ltd. Mice were housed in Allentown microisolation cages (4–6 mice per cage) or polysulfone individually ventilated cages (up to 5 mice per cage) with *ad libitum* access to food and water. Environmental conditions were maintained at 20°C to 26°C, 40% to 70% relative humidity, and a 12-hour light/dark cycle. Animals were monitored daily for morbidity, mortality, clinical signs, and abnormalities throughout the study.

To establish CDX models, 5 × 10^6^ PC-3-NECTIN4 or HT1376 cells (RRID: CVCL_1292) were subcutaneously implanted into the right flanks of male or female NU/NU mice, respectively. Tumors were allowed to grow to ∼190 mm^3^ (PC-3-NECTIN4) or ∼90 mm^3^ (HT1376) before treatment. For the MDA-MB-468 model, 1 × 10^7^ cells were implanted into the right flanks of female NOD/SCID mice, and tumors were grown to ∼110 mm^3^. Mice were randomized into groups (*n* = 7–8 per group) based on tumor volumes. Test drugs were administered intravenously in a single dose with a volume of 10 mL/kg on day 0. Studies were terminated on day 17 (PC-3-NECTIN4), day 14 (HT1376), and day 28 (MDA-MB-468).

The BL0597 (HuPrime) PDX model was developed by Crown Bioscience Inc. Tumor fragments (2–3 mm in diameter) were harvested from donor female BALB/c nude mice and subcutaneously implanted into the right upper flanks of recipient mice. Once tumors reached a mean volume of 137 mm^3^, 48 mice were randomized into groups based on tumor volumes (*n* = 8 per group). Treatments were administered intravenously on days 0, 7, and 14 in 0.9% saline at a dose volume of 10 mL/kg. The study was terminated on day 23.

Throughout the study, tumor volume and body weight were measured twice weekly. At study termination, all animals were euthanized, and tumors were excised and weighed. Antitumor efficacy was evaluated by comparing tumor volumes over time and final tumor weights between treatment and vehicle control groups. Tumor volume was calculated using the equation: V = 0.5 × L × W^2^, where L is tumor length and W is tumor width, measured using calipers. Percentage tumor growth inhibition (TGI) was calculated using the equation: $\text{TGI}\;=\;1\;-\;\left(T_{t}\;-\;T_{0}\right)/\left(V_{t}\;-\;V_{0}\right)]\;\times 100$, where T_0_ and T_t_ are the average tumor volumes of the treatment group on day 0 and at study termination, and V_0_ and V_t_ are the corresponding values for the vehicle control group, respectively.

### *In vitro* stability and *in vivo* PK

*In vitro* stability of CRB-701 and MMAE release was evaluated in human, monkey, and rat plasma, and in PBS with 0.1% Tween 20. Details are provided in Supplementary Methods. Blank human plasma was obtained from Tonghua Central Hospital and the drug clinical trial center of Hengyang Huacheng Hospital. Written informed consent was obtained from patients, studies were conducted in accordance with the Declaration of Helsinki, and the protocol was approved by appropriate institutional review boards.

Male and female Sprague–Dawley rats (Vital River Laboratory Animal Technology Co., Ltd; RRID: RGD_734476) and cynomolgus monkeys (Conghua Huazhen Animal Farm; *n* = 3 per sex per species) received a single intravenous infusion of 6 mg/kg CRB-701 or EV. In rats, a 5 mL/kg dose was infused over 2 minutes (2.5 mL/kg/minute); in monkeys, the same volume was infused over 20 minutes (0.25 mL/kg/minute).

For both species, blood samples were collected in potassium ethylenediaminetetraacetic acid tubes at the following time points: before the dose, end of infusion, and 4, 24, 72, 120, 168, 240, 336, 504, and 672 hours after the dose for PK bioanalysis.

An additional repeat-dose PK study was conducted in cynomolgus monkeys; one male animal and one female animal were administered weekly 6 mg/kg doses of CRB-701 or EV over a 22-day period. Blood samples were collected at day 1 (dose 1) and day 22 (dose 4) for PK bioanalysis.

### PK bioanalysis

Serum and tumor concentrations of CRB-701 and total antibody (tAb) in rat, mouse, and cynomolgus monkey samples were quantified using ELISAs. Free MMAE levels in plasma and tumor homogenates were measured by LC/MS-MS. All assays were validated and are described in detail in Supplementary Methods.

Concentration–time data for CRB-701, tAb, and MMAE were analyzed using Phoenix WinNonlin version 8.0 (Certara; RRID: SCR_024504). PK parameters, including maximum concentration (C_max_), time to maximum concentration (T_max_), half-life (T_1/2_), and area under the concentration–time curve (AUC), were calculated using noncompartmental analysis.

### Tumor uptake in xenograft models

The uptake of CRB-701 and free MMAE was evaluated in the MDA-MB-468 human breast cancer CDX model. NOD/SCID mice bearing tumors were randomized into five groups (*n* = 3 per time point per group) and administered a single intravenous dose of CRB-701 at 6 mg/kg. Groups were sacrificed at 0.5, 24, 96, 168, and 384 hours after the dose.

Serum samples were collected and centrifuged, and tumors were excised, homogenized in the presence of protease inhibitors, and centrifuged. Supernatants were extracted using methanol and acetonitrile for MMAE quantification by LC/MS-MS, and CRB-701 concentrations were determined from the homogenates by ELISA using standards spiked into tumor matrix. Detailed methodologies are provided in Supplementary Methods. Serum and tumor tissues from 15 untreated tumor-bearing animals served as blank controls.

### Toxicology studies

General toxicology studies were conducted at JOINN Laboratories, an Association for Assessment and Accreditation of Laboratory Animal Care International–accredited facility, in accordance with Good Laboratory Practice regulations and the International Council for Harmonisation S6, S9, and M3 (R2) guidelines. A 29-day repeat-dose toxicity study was conducted in male and female Sprague–Dawley rats to evaluate the safety profile of CRB-701 and determine the reversibility of any observed toxicities following a 4-week recovery period. Each dosing group included 10 rats per sex, with an additional 5 rats per sex per group in recovery cohorts. CRB-701 was administered via intravenous infusion at doses of 0, 5, 15, and 30 mg/kg once every 2 weeks for a total of three doses. A parallel 32-day repeat-dose toxicity study with a 4-week recovery period was conducted in cynomolgus monkeys (*n* = 3 per sex per group plus *n* = 2 per sex per group in recovery cohorts) with intravenous infusions of CRB-701 at 0, 2.5, 7.5, and 15 mg/kg every 2 weeks at a dosing volume of 10 mL/kg. Animals were dosed once every 2 weeks on days 1, 15, and 29 for a total of three doses.

Evaluations included clinical observations, body weight and food consumption, organ weights, macroscopic and microscopic pathology, ophthalmoscopic exams, clinical chemistry, hematology, coagulation, urinalysis, and toxicokinetic profiling of test article-related analytes (CRB-701, CRB-701-mAb, and free MMAE). In cynomolgus monkeys, additional safety pharmacology assessments were conducted to evaluate respiratory, cardiovascular, and central nervous system function.

### Statistical analysis

Tumor volume changes over time in CDX models were analyzed using one-way analysis of variance (ANOVA) with correction for multiple comparisons. For PDX models, tumor volume comparisons were performed using one-way ANOVA followed by *post hoc* Tukey or Dunnett tests, or the Kruskal–Wallis test where appropriate. Detailed statistical methodologies for PDX model analysis are provided in Supplementary Methods.

## Results

### Characterization of CRB-701

**Figure 1.**
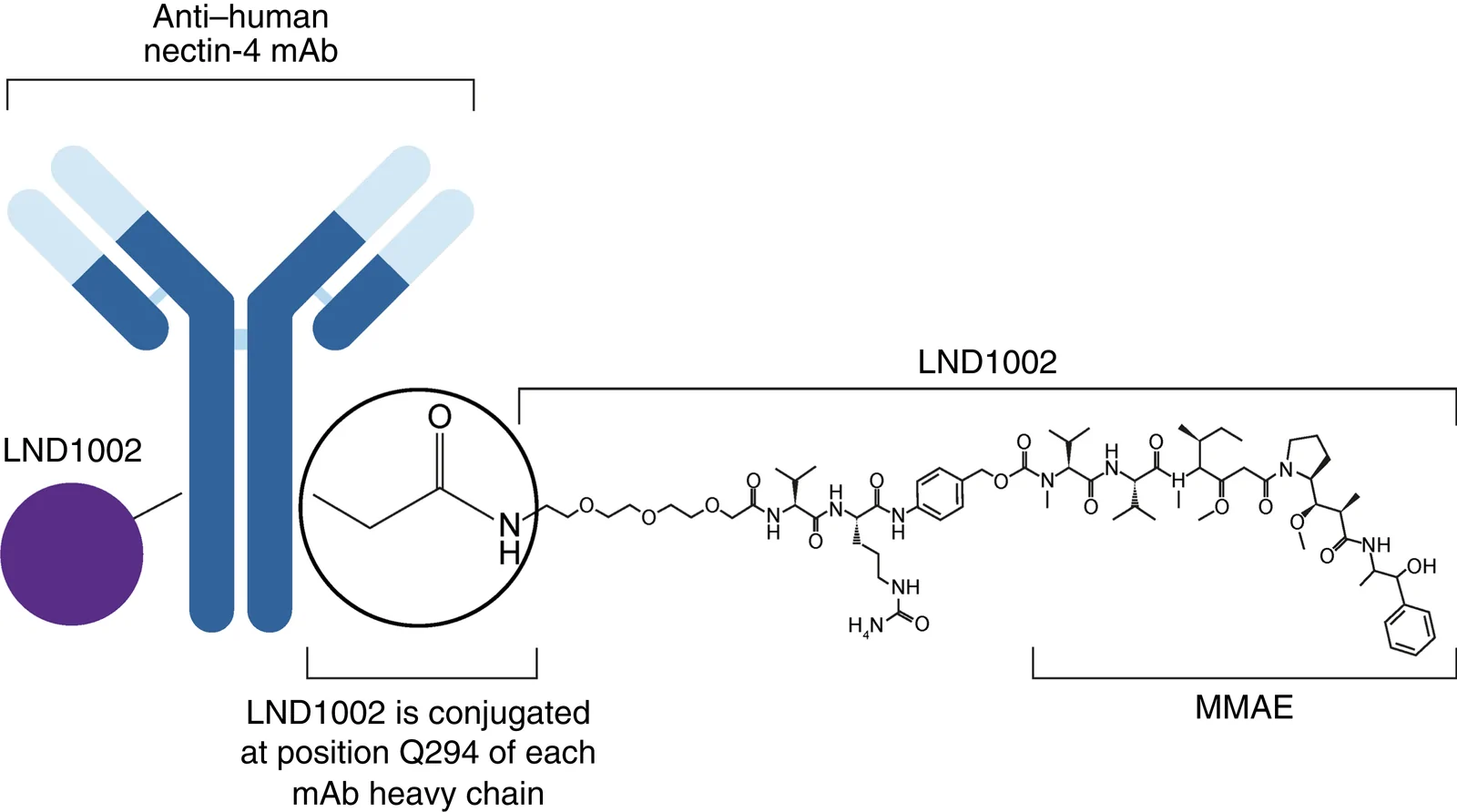
Structure of CRB-701. CRB-701 is comprised of a novel anti-human nectin-4 mAb conjugated to a molecule of LND1002 at the Q294 position of each heavy chain, yielding a mean DAR of 2. Each LND1002 molecule comprises a PEG moiety, a valine-citrulline *p*-aminobenzyl carbamate cathepsin B cleavable linker, and MMAE.

CRB-701 (Fig. 1) was characterized by size-exclusion chromatography and capillary electrophoresis under both reducing and nonreducing conditions, with all manufactured lots exhibiting high purity (>95%). Labeling was nearly quantitative, with ≤1.2% of antibody remaining unconjugated. The mean DAR, measured by reversed-phase high-performance liquid chromatography, was 2. Mass spectrometry indicated that the final product, CRB-701 heavy chain, had a molecular weight increased by 1,296.4 Da relative to the unlabeled antibody, consistent with the theoretical addition of one LND1002 molecule (1,295.6 Da), and was of high homogeneity, with >82.1% in the double conjugated form, 9.6% in the single conjugated form, and 8.1% in the triple conjugated form. Free MMAE or linker represented <0.0002% (w/w) of total mass.

### Nectin-4 binding activity

**Figure 2.**
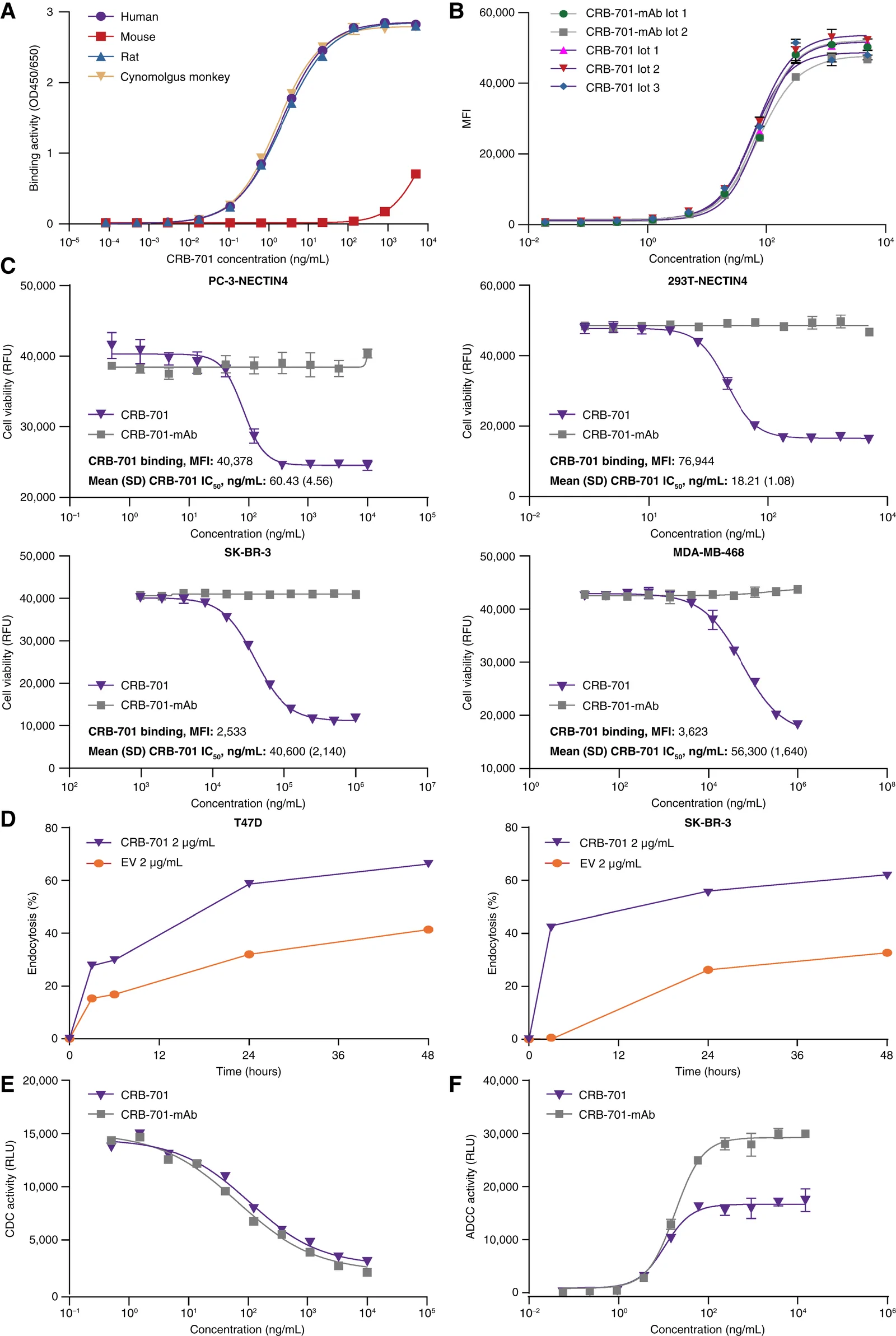
Characterization of CRB-701 and CRB-701-mAb via *in vitro* dose–response assays. **A,** Binding of CRB-701 to human, cynomolgus monkey, rat, and mouse nectin-4 proteins was assessed by ELISA. Absorbance was measured at 450/650 nm using a microplate reader as an indicator of binding activity. **B,** Binding of three independent lots of CRB-701 and two independent lots of CRB-701-mAb to PC-3-NECTIN4 cells assessed by flow cytometry. **C,** Cytotoxicity of CRB-701 and CRB-701-mAb in PC-3-NECTIN4, 293T-NECTIN4, SK-BR-3, and MDA-MB-468 cells. Cell viability was determined using a resazurin-based fluorescence assay. CRB-701 binding to nectin-4–expressing cells was determined by flow cytometry. **D,** Endocytosis of 2 μg/mL CRB-701 and 2 μg/mL EV in T47D and SK-BR-3 cells measured by confocal microscopy. Endocytosis percentages were calculated based on MFI increases upon internalization. **E,** CDC activity of CRB-701 and CRB-701-mAb in 293-NECTIN4 cells. CDC was determined using CellTiter-Glo Luminescent Cell Viability Assay following cell incubation with a human serum-derived complement. **F,** ADCC activity was tested using Jurkat/hFcγRIIIa-NFAT transgenic cells as the effector cells and the 293T-NECTIN4 cells as the target cells. ADCC was determined by luciferase assay; RLU indicates cytotoxic response. Graph data are presented as means ± standard deviation (SD) from three technical replicates at each concentration.

CRB-701 bound to human, rat, and monkey nectin-4 proteins similarly, with EC_50_ values of 2.01, 2.40, and 1.58 ng/mL, respectively (Fig. 2A). Binding activity to mouse nectin-4 was low with an EC_50_ greater than 5 μg/mL (31 nmol/L), the highest concentration tested. No binding was detected of CRB-701 to three human homologs of nectin-4: nectin-1, nectin-2, and nectin-3 at concentrations up to 5 μg/mL, demonstrating the high selectivity and specificity of the ADC (Supplementary Fig. S1). Binding of independent lots of CRB-701 or CRB-701-mAb to human nectin-4 was further assessed by BLI; a binding affinity (K_d_) of 2.06 to 2.19 nmol/L and 2.15 to 2.19 nmol/L (∼0.33 μg/mL) was determined for CRB-701 and CRB-701-mAb across the different lots, respectively, with no significant differences between the two (Supplementary Fig. S2).

Binding of independent lots of CRB-701 and CRB-701-mAb to PC-3-NECTIN4 cells was also assessed by flow cytometry (Fig. 2B); similar binding affinities were observed for the CRB-701-mAb [EC_50_, 77 ± 2.9 ng/mL (0.48 ± 0.018 nmol/L)] and CRB-701 [EC_50_, 67 ± 6.8 ng/mL (0.42 ± 0.042 nmol/L)]. Collectively, these results indicate that MMAE conjugation did not affect the nectin-4 binding activity of CRB-701-mAb.

### *In vitro* cytotoxicity

The ability of CRB-701 and CRB-701-mAb to inhibit cell survival was evaluated in nectin-4–transfected (PC-3-NECTIN4 and 293T-NECTIN4) and nontransfected breast cancer cell lines with endogenous levels of nectin-4 expression (SK-BR-3 and MDA-MB-468; Fig. 2C) and compared with EV in PC-3-NECTIN4 (Supplementary Fig. S3A) and MDA-MB-468 cells (Supplementary Fig. S3B). Flow cytometry analysis confirmed that the transfected cell lines expressed substantially higher levels of nectin-4, with CRB-701 binding (MFI) increased 10-fold in transfected cells versus nontransfected cells (Fig. 2C). The transfected cells were also approximately 1,000-fold more sensitive to the cytotoxic effects of CRB-701, reflecting the dependence of cytotoxicity on nectin-4 expression (mean IC_50_ in transfected cell lines, 18.2–60.4 ng/mL vs. 40,600–56,300 ng/mL in nontransfected cell lines). Compared with CRB-701, EV was more than threefold more potent in PC-3-NECTIN4 cells (Supplementary Fig. S3A), but potency was similar in MDA-MB-468 cells (Supplementary Fig. S3B). No cytotoxicity was observed in PC-3 (Supplementary Fig. S4A) or 293T (Supplementary Fig. S4B) parental cell lines treated with CRB-701 or EV at doses up to 1,667 or 883 ng/mL, respectively (Supplementary Fig. S4). As expected, the CRB-701-mAb had no effect on cell viability.

### Endocytosis, MMAE release, and bystander-mediated toxicity

In SK-BR-3 and T47D cell lines with endogenous levels of nectin-4 expression, CRB-701 uptake was rapid and consistently higher than EV uptake (approximately twofold at most time points) throughout the 48-hour time course (Fig. 2D).

MMAE conjugation did not affect endocytosis, with uptake of CRB-701 and CRB-701-mAb reaching 84% and 88% in PC-3-NECTIN4 cells after 48 hours, respectively (Supplementary Fig. S5). Following incubation with 2 μg/mL CRB-701, appreciable intracellular MMAE release was measured in PC-3-NECTIN4 cells (intracellular levels of 0.21 ng/mL at 6 hours and 0.55 ng/mL at 24 hours), whereas intracellular MMAE release was below the lower limit of quantification in nectin-4–negative PC-3 cells (Supplementary Table S2).

Consistent with previous reports characterizing intracellular release of MMAE from ADCs (23), CRB-701 induced cell-cycle arrest in 293T-NECTIN4 cells at the G_2_–M-phase, whereas CRB-701-mAb had no effect on cell-cycle distribution (Supplementary Fig. S6; Supplementary Table S3). Flow cytometry confirmed that CRB-701 resulted in large increases in the proportions of early- and late-stage apoptotic cells compared with controls (Supplementary Table S4). CRB-701 also induced dose-dependent activation of caspase 3/7 in PC-3-NECTIN4 cells (EC_50_ of 450.5 ± 20.3 ng/mL); CRB-701-mAb had little to no effect on apoptosis in either cell line (Supplementary Fig. S7; Supplementary Table S4).

Bystander-mediated toxicity was assessed using luciferase assays in cocultures of 293T-NECTIN4 or PC-3-NECTIN4 cells with nectin-4–negative HEK293-LUC cells (Supplementary Fig. S8A). CRB-701, but not CRB-701-mAb, induced dose-related reductions in the viability of nectin-4–negative HEK293-LUC cells, suggesting that MMAE diffuses into adjacent cells following its intracellular release (Supplementary Fig. S8B). Moreover, no cytotoxicity was observed in nontransfected PC-3 or 293T cells at doses up to 883 and 1,667 mg/mL, respectively (Supplementary Fig. S4A and S4B), which are concentrations that induce a maximum cell killing effect in the nectin-4–expressing cells. These results are consistent with the findings that nectin-4 expression is required for appreciable MMAE release from CRB-701 and that cytotoxicity in the bystander assay is not caused by MMAE spontaneously released in the supernatant.

### Effector activity: CDC and ADCC

The CRB-701 parent antibody is an IgG1 isotype antibody, and as such, both CRB-701 and CRB-701-mAb were expected to exert Fc-mediated effector functions on nectin-4–expressing cells. Consistent with this, both CRB-701 and CRB-701-mAb demonstrated CDC against 293T-NECTIN4 cells with similar potency (CRB-701 EC_50_: 105.4 ng/mL; CRB-701-mAb EC_50_: 66.7 ng/mL; Fig. 2E).

ADCC was evaluated using Jurkat/hFcγRIIIa-NFAT effector cells cocultured with 293T-NECTIN4 cells at a 15:1 effector:target ratio. Both CRB-701 and CRB-701-mAb induced ADCC responses, again with similar EC_50_ values (CRB-701 EC_50_: 19.1 ng/mL; CRB-701-mAb EC_50_: 18.8 ng/mL; Fig. 2F). However, a higher maximal ADCC response was observed with CRB-701-mAb than with CRB-701, suggesting that MMAE conjugation may cause a slight attenuation of the ADCC response.

These functional activities are supported by high-affinity binding of CRB-701 to FcγRIa (K_d_ of 4 nmol/L) and to C1q (K_d_ of 7 nmol/L); no obvious differences in Fc receptor binding affinity were observed between CRB-701 and CRB-701-mAb (Supplementary Table S5).

### *In vivo* efficacy

**Figure 3.**
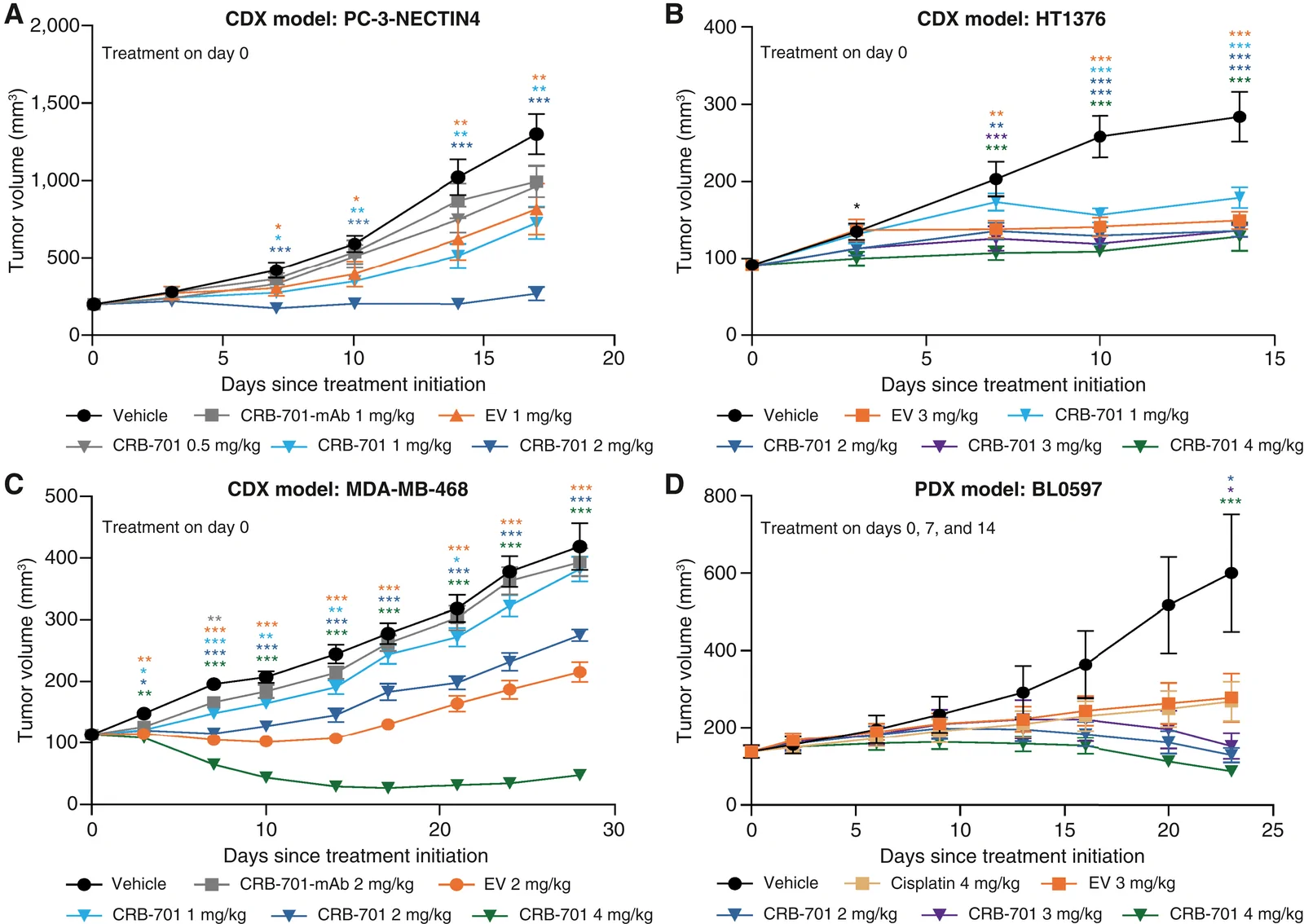
*In vivo* antitumor activity of CRB-701 in four xenograft mouse models. **A,** PC-3-NECTIN4 human prostate cancer CDX model (*n* = 7 per group). **B,** HT1376 human bladder cancer CDX model (*n* = 8 per group). **C,** MDA-MB-468 human triple-negative breast cancer CDX model (*n* = 8 per group). **D,** BL0597 human bladder cancer PDX model (*n* = 8 per group). Treatments were administered as a single intravenous dose on day 0 in CDX models. In the PDX model, treatments were administered once a week for 3 weeks. Tumor size measurements were taken twice a week and used to calculate tumor volumes. Data are presented as mean ± standard deviation (SD) for parts **A–C** and mean ± standard error of the mean (SEM) for part **D**; *, *P* < 0.05; **, *P* > 0.01; ***, *P* < 0.001 vs. vehicle. In the PDX model, CRB-701 4 mg/kg was significantly different (*P* = 0.0304) to EV 3 mg/kg.

CRB-701 demonstrated dose-dependent antitumor activity in three CDX models and one PDX model for human cancer (Fig. 3). These included the PC-3-NECTIN4 prostate cancer model that expresses high levels of nectin-4 (Fig. 2C) and three models with low-to-moderate endogenous nectin-4 expression: MDA-MB-468 (triple-negative breast cancer; low expression; Fig. 2C), HT1376 (bladder carcinoma; heterogeneous expression; ref. 24), and the PDX BL0597 (bladder cancer; low expression; ref. 25).

In the PC-3-NECTIN4 CDX model, a single 1 and 2 mg/kg dose of CRB-701 resulted in a mean TGI rate of 52.1% and 93.4% at study termination, respectively, versus a TGI of 44.1% with 1 mg/kg EV. A TGI rate of 27.8% was also observed with the CRB-701-mAb, possibly because of CDC or ADCC activity, which is still functional in NU/NU mice albeit without a T-cell component (Fig. 3A).

In the HT1376 CDX model, the mean TGI rates of 76.6% and 80.8% were observed with the 2 and 4 mg/kg doses of CRB-701, respectively, providing similar levels of antitumor activity to the 3 mg/kg dose of EV (TGI: 69.9%) at study termination (Fig. 3B).

In the MDA-MB-468 CDX model, 2 mg/kg CRB-701 produced a lower TGI versus 2 mg/kg EV (46.9% vs. 66.5%, respectively); however, the 4 mg/kg CRB-701 dose resulted in substantial tumor regression (TGI: 121.2%), indicating a dose-responsive effect, but with lower potency than the other models studied (Fig. 3C).

In the BL0597 PDX model, weekly intravenous administration of CRB-701 (2, 3, and 4 mg/kg every 3 weeks) inhibited tumor growth by 101.6%, 96.5%, and 110.7%, respectively (Fig. 3D). In contrast, 3 mg/kg EV (every 3 weeks) resulted in a TGI rate of 69.6%, suggesting that CRB-701 may have more potent antitumor activity to EV in tumors with low nectin-4 expression. The TGI difference versus 3 mg/kg EV was statistically significant at 4 mg/kg CRB-701 (*P* = 0.0304).

In the PC-3-NECTIN CDX model, the transplanted tumor had a negative impact on body weight that was significantly improved in all of the test drug treatment groups by study termination (*P* < 0.05 vs. vehicle; Supplementary Fig. S9A). CRB-701 treatment did not result in any significant changes in body weight in the HT1376, MDA-MB-468, and BL0597 models (Supplementary Fig. S9B–S9D).

### PK

*Ex vivo* stability studies demonstrated that CRB-701 is highly stable in human, monkey, and rat plasma at 37°C, with a half-life equal to or exceeding 770 hours (Supplementary Fig. S10; Supplementary Tables S6–S9). After 336 hours, released MMAE accounted for only ∼1% of the theoretical drug load, with similar release kinetics across species (Supplementary Fig. S6C), indicating minimal premature payload release.

**Figure 4.**
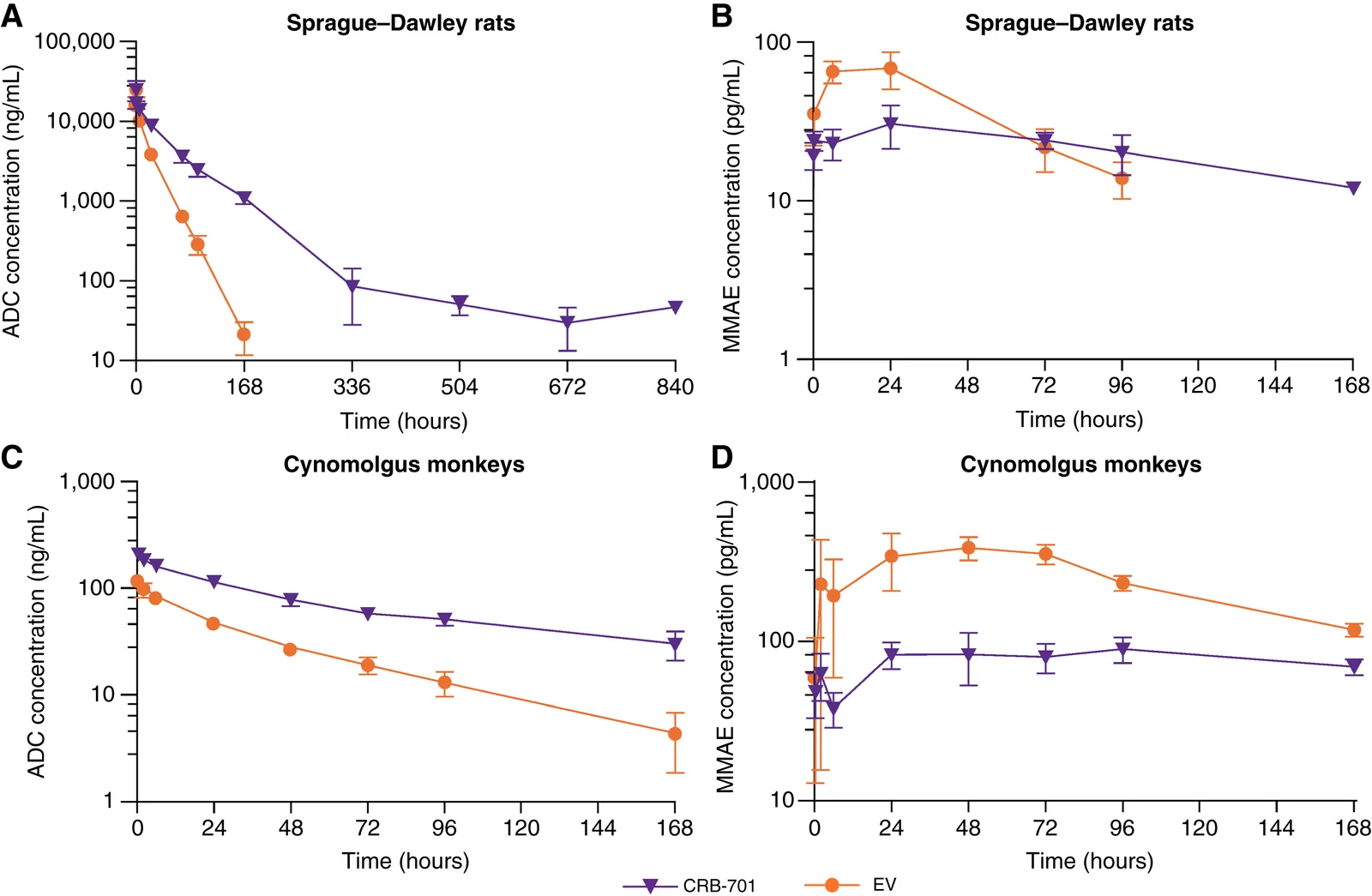
PK of CRB-701 and EV analytes, ADC (**A** and **C**), and MMAE (**B** and **D**) in plasma, following administration of 1 mg/kg CRB-701 or EV in Sprague–Dawley rats (*n* = 5), and 6 mg/kg CRB-701 or EV to cynomolgus monkeys (*n* = 2). ADC concentration was determined by ELISA, and MMAE concentration was determined by LC/MS-MS. Data are presented as mean ± standard deviation (SD).

PK profiles of CRB-701 and free MMAE were evaluated following a single intravenous dose of CRB-701 or EV of 1 mg/kg in Sprague–Dawley rats or 6 mg/kg in cynomolgus monkeys (Fig. 4A–D; Table 1). Compared with EV, CRB-701 exhibited an approximately fourfold longer mean half-life in rats (58 vs. 14.1 hours; Fig. 4C) and an approximately twofold longer mean half-life in monkeys (100 vs. 44.5 hours). This resulted in 3.4- and 2.9-fold higher AUC from dosing to the time of the last measured concentration (AUC_last_) in rats and monkeys, respectively. Although CRB-701 and EV C_max_ values were similar in rats, CRB-701 was associated with a 1.9-fold higher C_max_ than EV in monkeys.

**Table 1.** Mean PK of ADC, tAb, and MMAE analytes following a single intravenous dose of CRB-701 or EV in Sprague–Dawley rats and cynomolgus monkeys.

Parameter	CRB-701		EV	
	Rat	Monkey	Rat	Monkey
ADC	​	​	​	​
C_max_ (μg/mL)	24.9	210	24.5	112
AUC_last_ (hours × μg/mL)	931	11,761	274	4,116
T_1/2_ (hours)	58	100	14.1	44.5
tAb	​	​	​	​
C_max_ (μg/mL)	24.5	211	18.7	130
AUC_last_ (hours × μg/mL)	936	11,802	413	5,924
T_1/2_ (hours)	40	108	25.1	59.7
MMAE	​	​	​	​
C_max_ (pg/mL)	32.5	91	71.4	413
AUC_last_ (hours × pg/mL)	2,750	13,079	3,888	43,175
T_1/2_ (hours)	131	266	37.8	61.3
T_max_ (hours)	14.5	72.3	13.2	48.3

NOTE: Male Sprague–Dawley rats (*n* = 5) were treated with 1 mg/kg CRB-701 or EV; cynomolgus monkeys (*n* = 2) were treated with 6 mg/kg CRB-701 or EV. Concentrations of ADC and tAb analytes were determined by ELISA; MMAE analyte concentration was determined by LC/MS-MS. Parameters were derived from Phoenix WinNonlin noncompartmental fits to the data.

Importantly, systemic exposure to free MMAE was significantly lower with CRB-701 than with EV: C_max_ was 2.2-fold lower in rats (Fig. 4B) and 4.5-fold lower in monkeys (Fig. 3D), and AUC_last_ was 1.4- and 3.3-fold lower in rats and monkeys, respectively. These findings suggest that CRB-701 may be more stable in circulation, associated with lower systemic MMAE exposure but maintaining higher levels of antibody-bound drug than comparable doses of EV.

Similar results were also observed in a 22-day repeat-dose study in which monkeys received 6 mg/kg weekly doses of CRB-701 or EV, although this study was limited to one animal per sex. On day 22 (dose 4), CRB-701 ADC C_max_ and AUC_last_ were 1.3- and 1.8-fold higher than EV, respectively, whereas free MMAE C_max_ and AUC_last_ were 1.9- and 1.7-fold lower (Supplementary Table S10).

**Figure 5.**
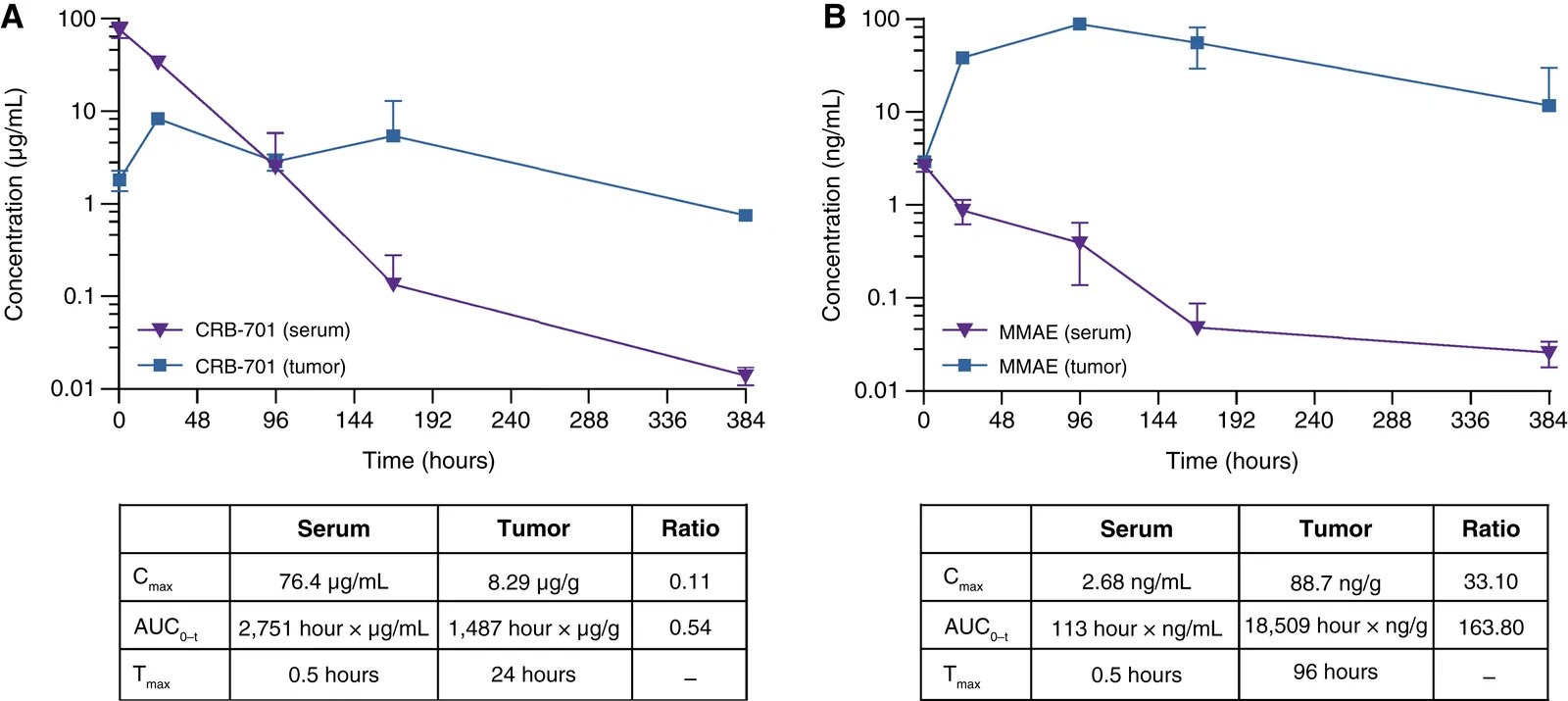
PK parameters and relative serum and tumor distribution of CRB-701 ADC (**A**) and free MMAE (**B**) analytes in the MDA-MB-468 human triple-negative breast cancer CDX model following a single intravenous injection of CRB-701 at 6 mg/kg. Data are presented as mean ± standard deviation (SD) from three mice per time point. Ratio refers to the tumor:serum ratio.

Tissue distribution studies in mice bearing MDA-MB-468 xenografts further supported the selective tumor targeting of CRB-701 (Fig. 5; Supplementary Table S11). Following a single 6 mg/kg dose, CRB-701 rapidly accumulated in tumors, reaching peak levels at 24 hours and remaining detectable throughout the 14-day study (Fig. 5A). Systemic concentration was initially 42-fold higher than tumor levels, and with CRB-701 clearance from the serum, the tumor:serum ratio increased to >38 at 168 hours, reflecting sustained tumor retention.

Free MMAE peaked in plasma at 0.5 hours after the dose and declined steadily, whereas tumor concentrations peaked at 96 hours (Fig. 5B). Notably, intratumoral MMAE exposure [C_max_ and AUC from 0 hours to the last measurable concentration time point (AUC_0–t_)] was 33- and 164-fold higher, respectively, than systemic levels, indicating tumor-selective targeting of the MMAE payload following CRB-701 treatment (Fig. 5B).

### Toxicology

The severely toxic dose in 10% of animals (STD10) was determined to be 30 mg/kg in rats after 28 days of every 2 weeks of dosing, whereas the highest nonseverely toxic dose (HNSTD) in cynomolgus monkeys was 7.5 mg/kg.

In rats, administration of three doses of 30 mg/kg CRB-701 resulted in treatment-related toxicities affecting the eyes, pituitary gland, testes, epididymides, prostate gland, male mammary gland, ovaries, oviducts, and liver. Hematologic toxicity and immune system suppression were also observed. Four weeks after dosing was completed, evidence of recovery or partial resolution was observed across most organ types, with the exception of the male mammary gland, testes, epididymides, and prostate gland.

In monkeys, one male animal in the high-dose group (15 mg/kg) was found dead on day 42, which was attributed to systemic infection secondary to progression of infection at a skin lesion. The HNSTD was 7.5 mg/kg and was associated with reductions in red blood cell indices (red blood cell count, hemoglobin, and hematocrit) and white blood cell counts (total white blood cells, lymphocytes, and neutrophils). Histopathologic findings in doses up to 7.5 mg/kg included lesions of the skin, liver, thymus, bone marrow (sternum), eyes (cornea/conjunctiva), and mucosal tissues of the esophagus, larynx, tongue, and vagina. Skin toxicity consisted of epidermal hyperplasia, increased mitoses, and/or increased apoptosis of epidermal epithelium, which were minimal to slight in severity at this dose (Supplementary Table S12). Similar findings were noted in the adnexal/conjunctival epithelium of the eyes at 15 mg/kg and in one male at 7.5 mg/kg. The microscopic changes in affected organs were largely reversible, with most findings showing complete or partial resolution after a 4-week recovery period.

No toxicologically significant CRB-701-related effects were observed on body weight, oxygen saturation, coagulation parameters, urinalysis, or T-cell subsets. Additionally, dedicated assessments of the central nervous, cardiovascular, and respiratory systems, conducted as part of this study, revealed no toxicities.

Key toxicokinetic parameters (C_max_, AUC_last_, and C_ave_) after single and multiple doses for CRB-701 are provided in Supplementary Table S13 and are compared with published values for EV (17). Although data obtained after multiple doses must be taken with caution because of possible interference from antidrug antibodies, the data on day 1 indicate that exposures at the highest tolerated doses were higher for CRB-701 than for EV. Dose-normalized C_ave_ on day 1 values were similar, indicating that the longer half-life of CRB-701 provided similar average daily plasma concentrations to once-weekly EV.

## Discussion

Here, we describe development and characterization of CRB-701, a novel next-generation anti–nectin-4 ADC. Similar to other nectin-4–targeted agents (24, 26, 27), MMAE is conjugated to the parent antibody via a valine-citrulline cathepsin-B cleavable linker. However, in contrast to the maleimide-based cysteine conjugation used to synthesize EV and other ADCs (28), CRB-701 used a proprietary mTGase platform to conjugate MMAE to two specific glutamine residues within the IgG dimer. Maleimide-based cysteine conjugation typically yields a heterogeneous mixture of DARs, which may affect PK, stability, and efficacy (28); here, we demonstrate that the mTGase-based site-specific approach delivers a relatively homogeneous, highly enriched ADC product with high purity and a mean DAR of 2. In prolonged circulation, maleimide-based linkers are also prone to deconjugation via reverse Michael addition, which may result in off-target MMAE release and could diminish antitumor activity (18, 29). The mTGase-mediated isopeptide bond is inherently chemically stable (30) and aims to overcome this limitation.

*In vitro* characterization studies indicate that CRB-701 functions as designed, exhibiting nectin-4-dependent cellular uptake, intracellular MMAE release, and cytotoxicity. In cocultures of nectin-4-positive and -negative cells, CRB-701 also mediated bystander cytotoxicity—an important property for efficacy given the heterogeneity of human tumors. CRB-701 also retains ADCC and CDC activity, which could enhance the antitumor innate immune response, particularly if used in combination with checkpoint inhibitors that provide complementary (adaptive immune) activation mechanisms (31). Further research is warranted to validate these effects in fully immune-competent models and in human clinical settings.

The tumor-selective cytotoxicity demonstrated *in vitro* was also confirmed in CDX models; PK studies demonstrated substantial accumulation of free MMAE within the tumor microenvironment, with relatively low levels released in circulation. In line with this, CRB-701 exhibited potent antitumor activity in three CDX models for prostate, breast, and bladder cancers and in a PDX model of bladder cancer with varying levels of nectin-4 expression. In the nectin-4–overexpressing PC-3-NECTIN4 prostate cancer CDX model, CRB-701 achieved 93.4% TGI relative to vehicle control at the 2 mg/kg dose. In the bladder cancer model with lower (and heterogeneous) levels of nectin-4 expression, the 2 mg/kg dose was associated with 76.6% TGI. In both the prostate and bladder cancer CDX models, a single dose of CRB-701 exhibited similar efficacy to EV at the equivalent single dose, despite differing nectin-4 expression levels. The breast cancer model was slightly less responsive to the 2 mg/kg CRB-701 dose than the other CDX models, but this dose was nevertheless similar in efficacy to 2 mg/kg EV, whereas 4 mg/kg CRB-701 induced substantial tumor regression (TGI: 121.2%). A weekly dosing regimen in the PDX model resulted in robust TGI at 2 to 4 mg/kg doses (TGI: 96.5% to 110.7%), which was greater than the response achieved with weekly 3 mg/kg doses of EV (TGI: 69.6%). Despite the limitations of *in vitro* cytotoxicity assays to predict *in vivo* activity (drug clearance, *in vivo* tumor penetration, and uptake are not accounted for), *in vivo* activity was consistent with *in vitro* cytotoxicity results in which EV exhibited similar potency to CRB-701 (threefold higher potency in the PC-3-NECTIN4 cell line and very similar potency in the MDA-MB-468 cells).

These mouse xenograft results suggest that CRB-701 delivers a potent intratumoral dose of MMAE that is sufficient to block tumor progression to a similar or greater extent than EV, despite its lower DAR. This may be in part due to the higher rate of cellular uptake compared with EV observed in cell line models. Higher DARs are not necessarily advantageous; if the level of intracellular MMAE release exceeds the level needed to induce cellular apoptosis, excess MMAE may be released into the periphery, increasing the likelihood of off-target toxicity. A high DAR may also result in more potent cytotoxicity toward nectin-4–expressing healthy cells, which could further compromise the drug safety profile. The lower MMAE drug load in CRB-701 reduces overall hydrophobicity, a property that is associated with slower clearance and an improved PK profile in ADCs (32). Indeed, CRB-701 exhibits a substantially longer half-life than EV and provides an extended duration of tumor delivery, which may partly explain why similar antitumor activity was observed with EV in single-dose studies, despite differences in *in vitro* potency. A longer half-life may also enable less frequent clinical dosing than the weekly dosing required for EV administration. Extrapolation of monkey PK data suggests that every 2 weeks or every 3 weeks CRB-701 dosing intervals may be feasible in humans, potentially offering greater convenience for patients.

PK studies in rats and nonhuman primates also revealed that CRB-701 resulted in lower systemic exposure to MMAE compared with EV, which may lower the risk of MMAE-related toxicities such as peripheral neuropathy, a condition linked to nonspecific uptake of this microtubule disruptor in nerve tissue (6). Peripheral neuropathy occurred in 53% of patients treated with EV monotherapy, 30% of patients experienced grade 2 reactions, and 5% experienced grade 3 to 4 reactions. These adverse events resulted in dose interruptions or reductions when symptoms worsened, as well as discontinuations in 6% of patients (17). Reduced MMAE in circulation may alleviate this significant off-target treatment complication.

CRB-701 exhibited an improved nonclinical safety profile in GLP repeat-dose toxicology studies compared with EV. The STD10 for CRB-701 in rats was 30 mg/kg after four every-2-weeks doses, whereas EV exhibited adverse toxicities including one death at 10 mg/kg following once-weekly dosing (17). At these respective doses, CRB-701 average plasma concentration was approximately 2.8- to 7-fold higher. The HNSTD for CRB-701 in monkeys was 7.5 mg/kg after three every-2-weeks doses; for EV, a dose of just 6 mg/kg resulted in mortality, and 3 mg/kg was identified as a no observed adverse effect level. Mortality in the EV primate study was due to severe skin lesions in 3 of 10 animals on days 11 to 13, after administration of a second dose on day 8 (33). By comparison, one monkey (of 10) receiving 15 mg/kg CRB-701 died because of a skin infection on day 42, whereas skin lesions at 7.5 mg/kg were minimal to slight in severity. Skin reactions affect around half of patients receiving EV, with around 13% of patients experiencing grade 3 or 4 events, an on-target effect owing to nectin-4 expression in skin tissue (34). Comparing exposure at the respective tolerable doses, CRB-701 average plasma concentration was approximately 2.9- to 8.8-fold higher than EV, consistent with the improvement in tolerability for CRB-701. The lower severity and incidence of skin toxicities seen for CRB-701 has the potential to translate to an improved safety margin for this toxicity linked to nectin-4–mediated uptake. Overall, further clinical characterization of CRB-701 is warranted to determine if skin lesions and peripheral neuropathy may be less of a concern for CRB-701 versus EV, given the lower DAR and favorable ADC linker stability.

Beyond CRB-701, other approaches for improving ADC linker stability using mTGase-mediated conjugation have yielded similar benefits. For example, employment of a stable peptide-based linker resulted in an anti-CD79b–targeted ADC that demonstrated potent TGI with minimal free MMAE in rodent models, achieving an improved therapeutic index over the comparator ADC, polatuzumab vedotin (8). These findings provide further validation for the use of site-specific, stable linker conjugation chemistries to optimize ADC efficacy and safety.

Collectively, the preclinical toxicology and PK results, in which higher doses and exposures were tolerated in rats and cynomolgus monkeys, together with improved response in the PDX model at an equivalent dose to EV, suggest that CRB-701 may represent a promising alternative to EV for the treatment of nectin-4-positive tumors. Although confirmation in human studies is needed, these preclinical findings suggest potential for an improved safety margin, supporting exploration of higher doses and evaluation in tumor types less responsive to EV.

CRB-701 is under investigation in a phase I dose-escalation study in China, assessing both every-2-weeks and every-3-weeks dosing schedules, and in the United States and Europe (NCT06265727), in which every-3-weeks dosing is under investigation. Preliminary data from these trials are encouraging and further support these preclinical findings, demonstrating early signs of antitumor activity in metastatic urothelial, head and neck, cervical, and endometrial cancers, alongside a favorable tolerability profile (35–37). These results suggest that CRB-701 has potential to offer meaningful clinical benefit across a range of nectin-4–expressing malignancies.

## Supplementary Material

Supplementary Methods Supplementary Methods

Figure S1 Figure S1. (?):ELISA measuring binding of CRB-701 to His-tagged nectin-1, nectin-2, nectin-3, nectin-4, and PVR.

Figure S2 Figure S2. (A) Representative binding curves of interaction between CRB-701-mAb and nectin-4 (fitted to Kd: 2.193 nM; r2: 0.9977) and (B) CRB-701 and nectin-4 (fitted to Kd: 2.063 nM; r2: 0.9964) as determined by the BLI method.

Figure S3 Figure S3. Cytotoxicity of CRB-701, CRB-701-mAb, and EV in (A) PC-3-NECTIN4 and (B) MDA-MB-468 cells. Cell viability was determined using a resazurin-based fluorescence assay.

Figure S4 Figure S4. Cytotoxicity of CRB-701, CRB-701-mAb, and EV in (A) PC-3 and (B) 293T cells. Cell viability was determined using a resazurin-based fluorescence assay.

Figure S5 Figure S5. Endocytosis of 2 μg/mL CRB-701 or CRB-701-mAb in PC-3-NECTIN4 cells assessed by flow cytometry. Endocytosis percentages were calculated based on MFI reduction.

Figure S6 Figure S6. Cell cycle analysis of 293T-NECTIN4 cells by flow cytometry using PI staining. Cells were treated with (A) blank control (cell culture medium), (B) 10 μg/mL CRB-701-mAb or (C) 10 μg/mL CRB-701 for 66 ± 3 hours. Fluorescence intensity histograms were fitted to a cell cycle distribution model based on chromosomal content: G1 phase (2N), S phase (2N–4N), and G2/M phase (4N).

Figure S7 Figure S7. Effect of CRB-701 and CRB-701-mAb on caspase-3/7 activity in PC-3-NECTIN4 cells measured with a Caspase-Glo 3/7 Assay Kit.

Figure S8 Figure S8. (A) Bystander-mediated cytotoxicity of CRB-701 or CRB-701-mAb in 293T-NECTIN4 and PC-3-NECTIN4 cells as determined by luciferase assay. A reduction in luminescence indicates a reduction in the viability of cocultured HEK293-LUC cells. Data are presented as mean ±SDs of three technical replicates. (B) Dose-response analysis of CRB-701 and CRB-701-mAb bystander-mediated cytotoxicity in PC-3-NECTIN4 cells.

Figure S9 Figure S9. Body weight changes by treatment groups in four xenograft mouse models.

Figure S10 Figure S10. Plasma stability of CRB-701 and CRB-701-mAb, and MMAE release from CRB-701.

Table S1 Table S1. List of key materials, suppliers, and (where applicable) RRIDs

Table S2 Table S2. Intracellular MMAE release in PC-3 and PC-3-NECTIN4 cells following treatment with 2 μg/mL CRB-701

Table S3 Table S3. Effect of CRB-701-mAb and CRB-701 on 293T-NECTIN4 cell cycle distribution

Table S4 Table S4. Apoptosis induction in 293T-NECTIN4 cells following treatment with 10 μg/mL CRB-701 or CRB-701-mAb, or vehicle

Table S5 Table S5. Binding affinities of CRB-701 and CRB-701-mAb to human Fc receptors and C1q determined by BLI

Table S6 Table S6. Stability of CRB-701 (0.02, 0.2, or 2 μM) over time following incubation with human, cynomolgus monkey, or Sprague Dawley rat plasma, or PBST control

Table S7 Table S7. Stability of CRB-701-mAb (0.02, 0.2, or 2 μM) over time following incubation with human, cynomolgus monkey, or Sprague Dawley rat plasma, or PBST control

Table S8 Table S8. Mean MMAE release (%) over time following incubation of CRB-701 (0.02, 0.2, or 2 μM) with human, cynomolgus monkey, or Sprague Dawley rat plasma, or PBST control

Table S9 Table S9. Half-life of CRB-701 (0.02, 0.2, or 2 μM) following incubation in human, cynomolgus monkey, or Sprague Dawley rat plasma

Table S10 Table S10. Mean toxicokinetic parameters at day 1 and day 22 for CRB-701 and EV analytes (ADC, tAb, and MMAE) following four weekly doses of 6 mg/kg CRB-701 or EV in cynomolgus monkeys

Table S11 Table S11. Serum and tumor concentrations of CRB-701 and MMAE over time in the MDA-MB-468 human triple-negative breast cancer CDX model following a single 6 mg/kg dose of CRB-701.

Table S12 Table S12. Skin toxicity events in cynomolgus monkey

Table S13 Table S13. Toxicokinetic exposures from 4-week repeat dose GLP toxicology studies in the rat and in cynomolgus monkeys following single and repeat doses of CRB-701 on Day 1 and Day 29 and EV on Day 1 and Day 22.

## Data Availability

The data generated in this study are available upon reasonable request to the corresponding author.
