# Supplementary material for: CRB-701: A Second-generation Nectin-4–Targeted Antibody–Drug Conjugate with Optimized Stability and Pharmacokinetics for the Treatment of Solid Tumors
Source: Cancer Res Commun. 2026 Sep 15;6(9):2164–76. doi: 10.1158/2767-9764.CRC-25-0799 (PMC13576400; doi:10.1158/2767-9764.CRC-25-0799)
Supplement: Supplementary file 1 [file crc-25-0799_figure_s10_suppsf10.pdf]

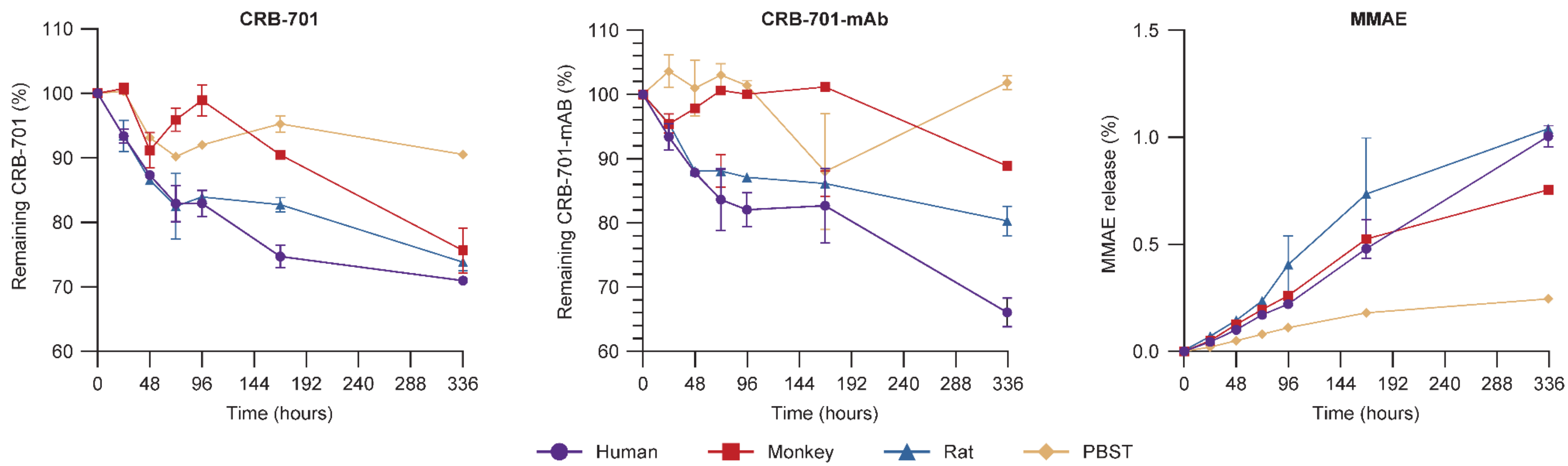

Figure S10. Plasma stability of CRB-701 and CRB-701-mAb, and MMAE release from CRB-701. CRB-701 or CRB-701-mAb (2 μM) was incubated with human, cynomolgus monkey, or Sprague Dawley rat plasma, or PBST control for 336 hours at 37°C. CRB-701 and CRB-701-mAb levels were determined by ELISA and MMAE levels were determined by LC-MS/MS. Remaining CRB-701 and CRB-701-mAb levels are shown as the proportion of the total concentration at time point 0. MMAE release is the measured MMAE concentration shown as a percentage of the theoretically expected molality.

ELISA, enzyme-linked immunosorbent assay; LC-MS/MS, liquid chromatography–tandem mass spectrometry; mAb, monoclonal antibody; MMAE, monomethyl auristatin E; PBST, phosphate-buffered saline with 0.1% Tween-20.
