# Supplementary material for: CRB-701: A Second-generation Nectin-4–Targeted Antibody–Drug Conjugate with Optimized Stability and Pharmacokinetics for the Treatment of Solid Tumors
Source: Cancer Res Commun. 2026 Sep 15;6(9):2164–76. doi: 10.1158/2767-9764.CRC-25-0799 (PMC13576400; doi:10.1158/2767-9764.CRC-25-0799)
Supplement: Supplementary file 2 [file crc-25-0799_figure_s1_suppsf1.pdf]

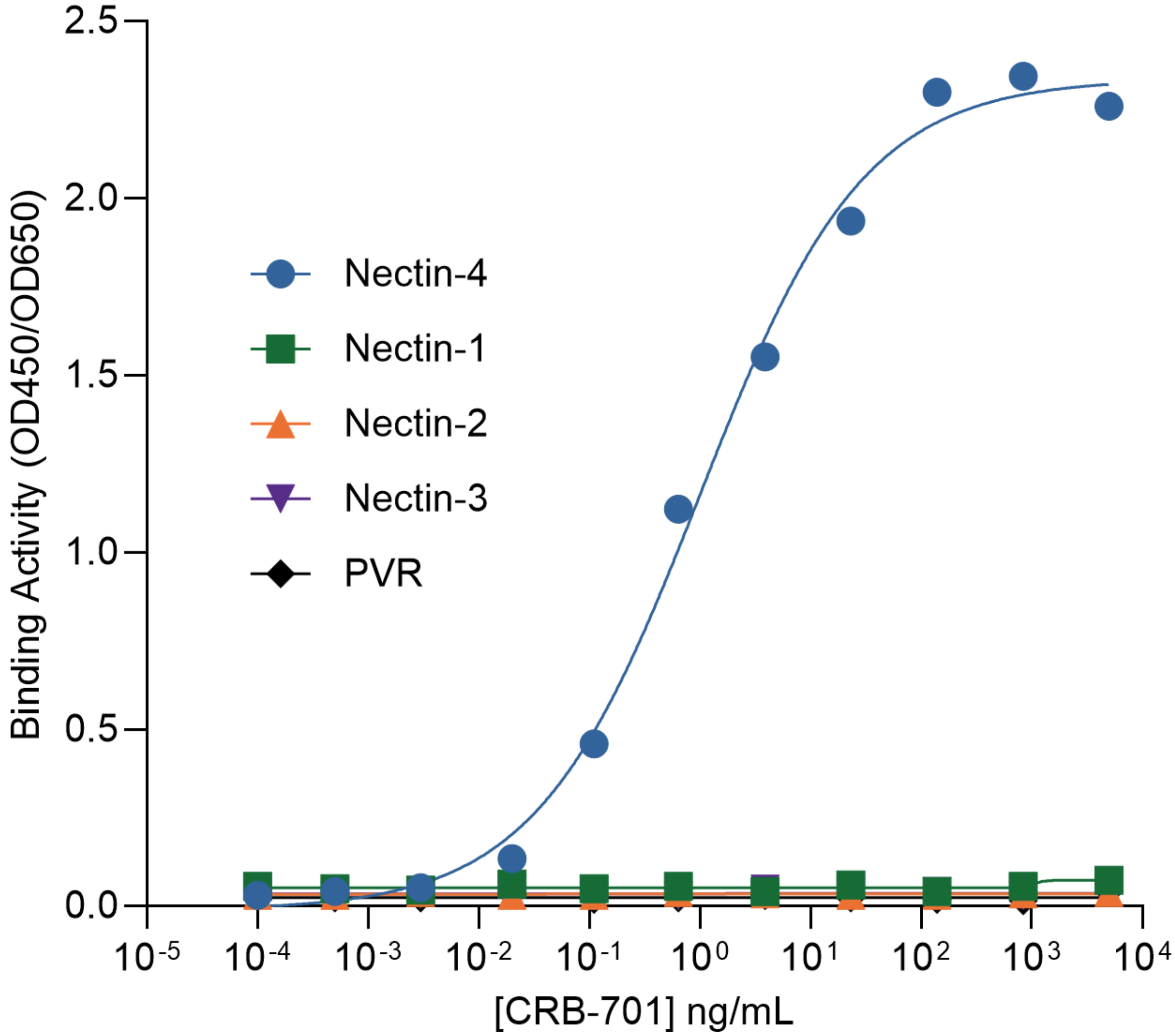

Figure S1. (?):ELISA measuring binding of CRB-701 to His-tagged nectin-1, nectin-2, nectin-3, nectin-4, and PVR. Data are average of n=3 replicate wells. Data fit to a four-parameter binding model for nectin-4 indicated an ED_{50} of 0.97 ng/mL; no appreciable binding was detected for CRB-701 to nectin-1, nectin-2, nectin-3, and PVR.

ELISA, enzyme-linked immunosorbent assay
