# Supplementary material for: CRB-701: A Second-generation Nectin-4–Targeted Antibody–Drug Conjugate with Optimized Stability and Pharmacokinetics for the Treatment of Solid Tumors
Source: Cancer Res Commun. 2026 Sep 15;6(9):2164–76. doi: 10.1158/2767-9764.CRC-25-0799 (PMC13576400; doi:10.1158/2767-9764.CRC-25-0799)
Supplement: Supplementary file 3 [file crc-25-0799_figure_s2_suppsf2.pdf]

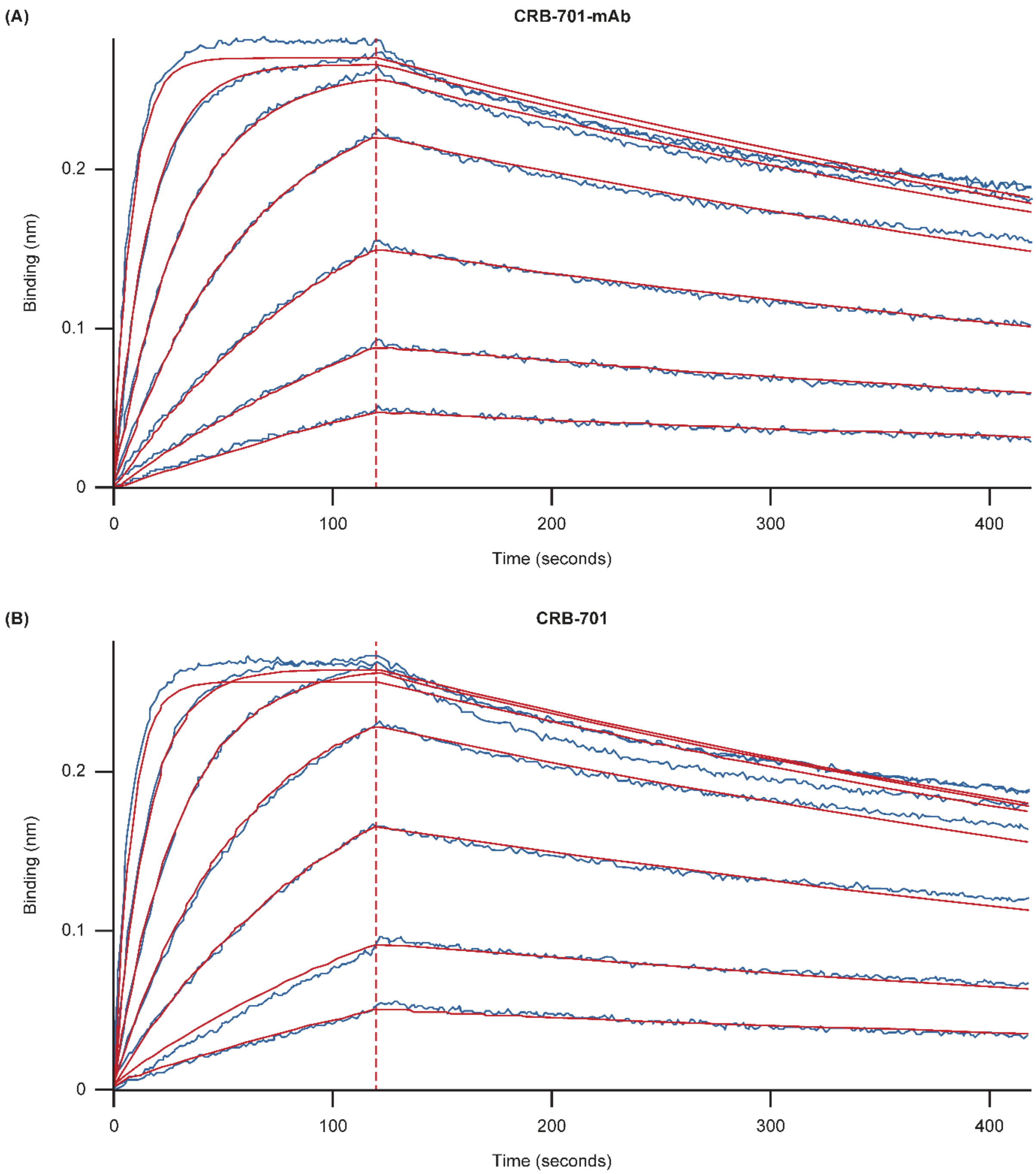

Figure S2. (A) Representative binding curves of interaction between CRB-701-mAb and nectin-4 (fitted to K_d: 2.193 nM; r^2: 0.9977) and (B) CRB-701 and nectin-4 (fitted to K_d: 2.063 nM; r^2: 0.9964) as determined by the BLI method.

BLI, biolayer interferometry; K_d, binding affinity (koff/kon); mAb, monoclonal antibody.
