# Supplementary material for: CRB-701: A Second-generation Nectin-4–Targeted Antibody–Drug Conjugate with Optimized Stability and Pharmacokinetics for the Treatment of Solid Tumors
Source: Cancer Res Commun. 2026 Sep 15;6(9):2164–76. doi: 10.1158/2767-9764.CRC-25-0799 (PMC13576400; doi:10.1158/2767-9764.CRC-25-0799)
Supplement: Supplementary file 4 [file crc-25-0799_figure_s3_suppsf3.pdf]

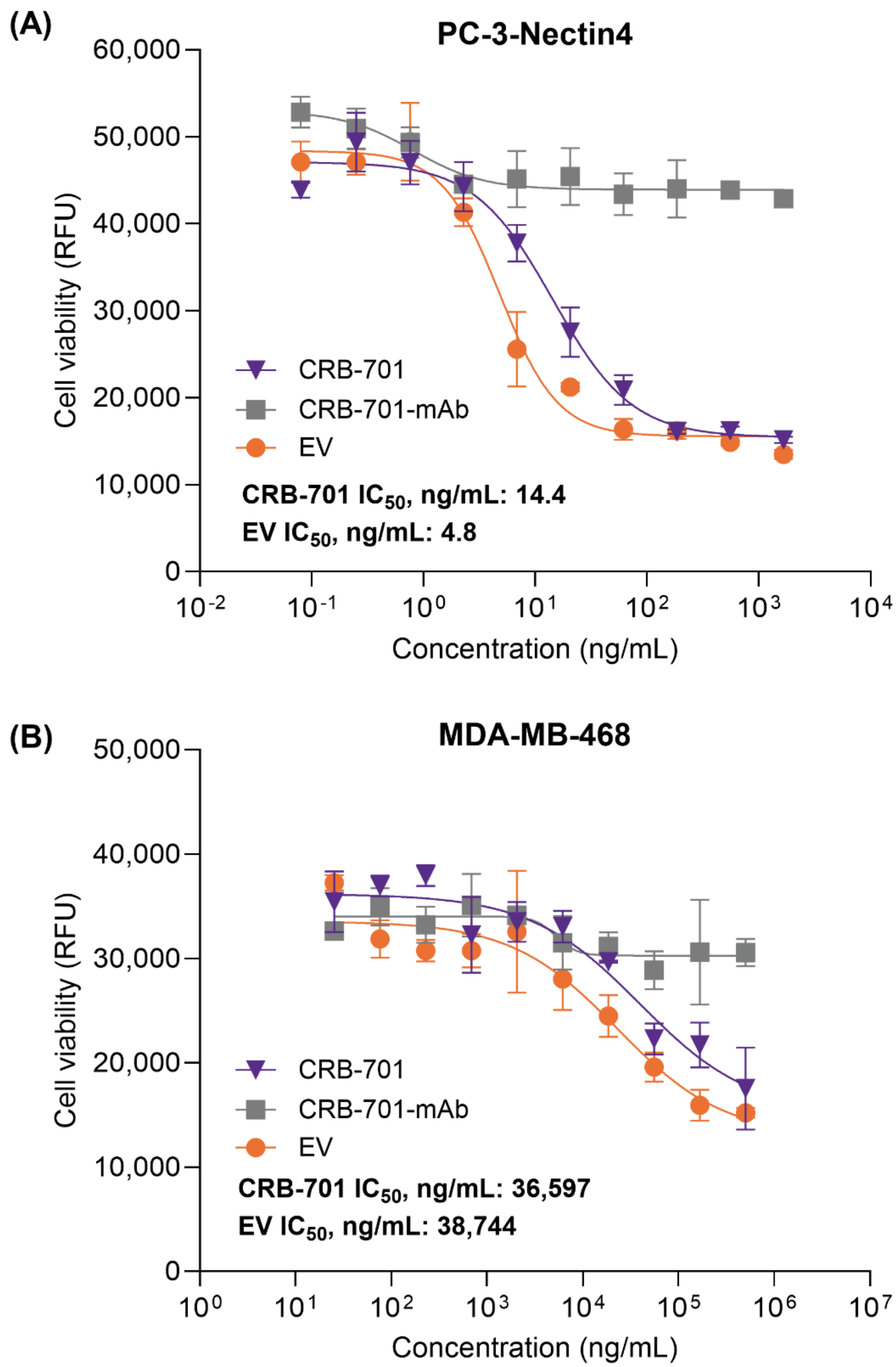

Figure S3. Cytotoxicity of CRB-701, CRB-701-mAb, and EV in (A) PC-3-NECTIN4 and (B) MDA-MB-468 cells. Cell viability was determined using a resazurin-based fluorescence assay.

EV, enfortumab vedotin; IC_{50}, concentration required to produce 50% maximal inhibition; mAb, monoclonal antibody; RFU, relative fluorescence unit.
