# Supplementary material for: CRB-701: A Second-generation Nectin-4–Targeted Antibody–Drug Conjugate with Optimized Stability and Pharmacokinetics for the Treatment of Solid Tumors
Source: Cancer Res Commun. 2026 Sep 15;6(9):2164–76. doi: 10.1158/2767-9764.CRC-25-0799 (PMC13576400; doi:10.1158/2767-9764.CRC-25-0799)
Supplement: Supplementary file 6 [file crc-25-0799_figure_s5_suppsf5.pdf]

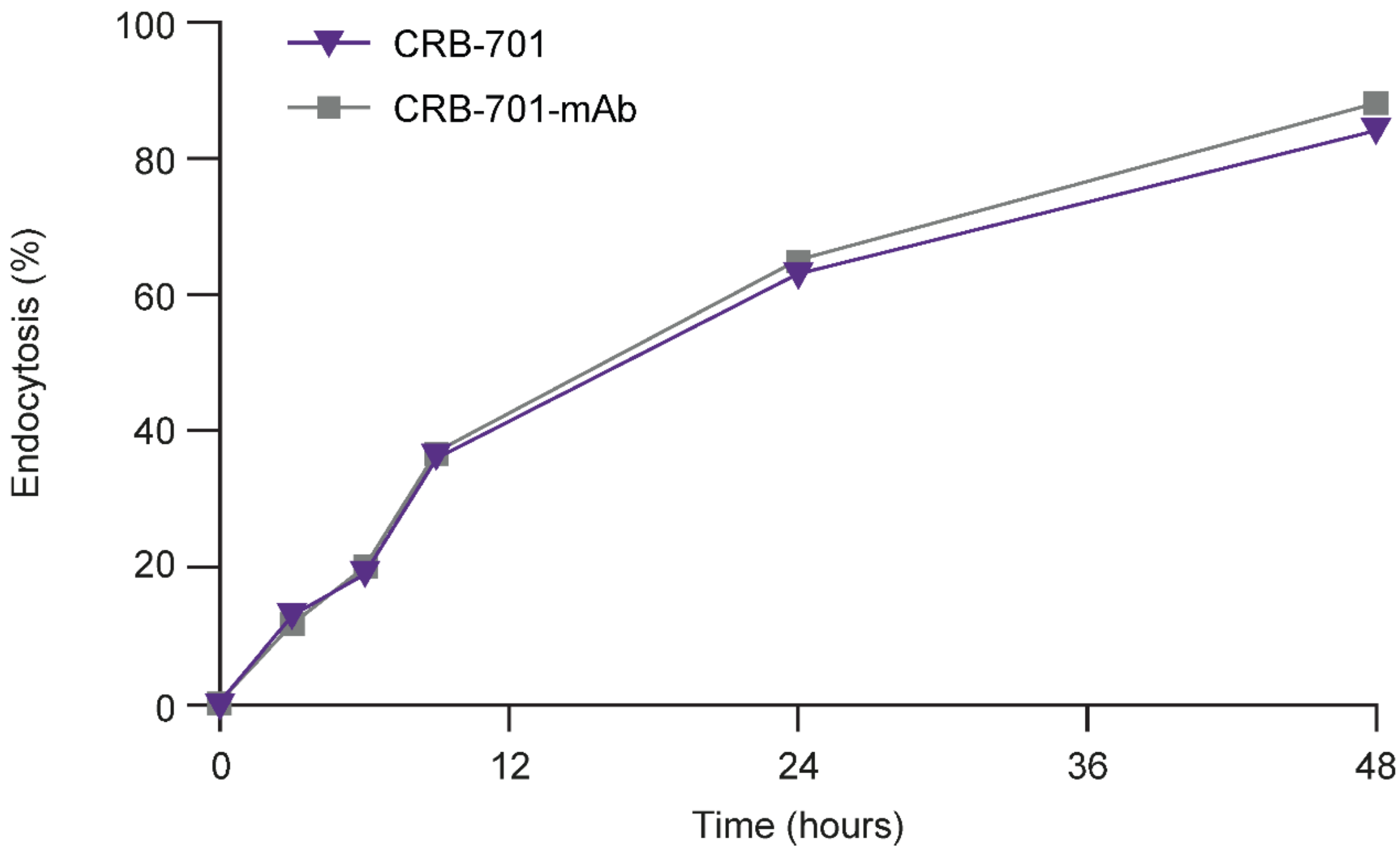

Figure S5. Endocytosis of 2 µg/mL CRB-701 or CRB-701-mAb in PC-3-NECTIN4 cells assessed by flow cytometry. Endocytosis percentages were calculated based on MFI reduction.

mAb, monoclonal antibody; MFI, median fluorescence intensity.
