# Supplementary material for: CRB-701: A Second-generation Nectin-4–Targeted Antibody–Drug Conjugate with Optimized Stability and Pharmacokinetics for the Treatment of Solid Tumors
Source: Cancer Res Commun. 2026 Sep 15;6(9):2164–76. doi: 10.1158/2767-9764.CRC-25-0799 (PMC13576400; doi:10.1158/2767-9764.CRC-25-0799)
Supplement: Supplementary file 7 [file crc-25-0799_figure_s6_suppsf6.pdf]

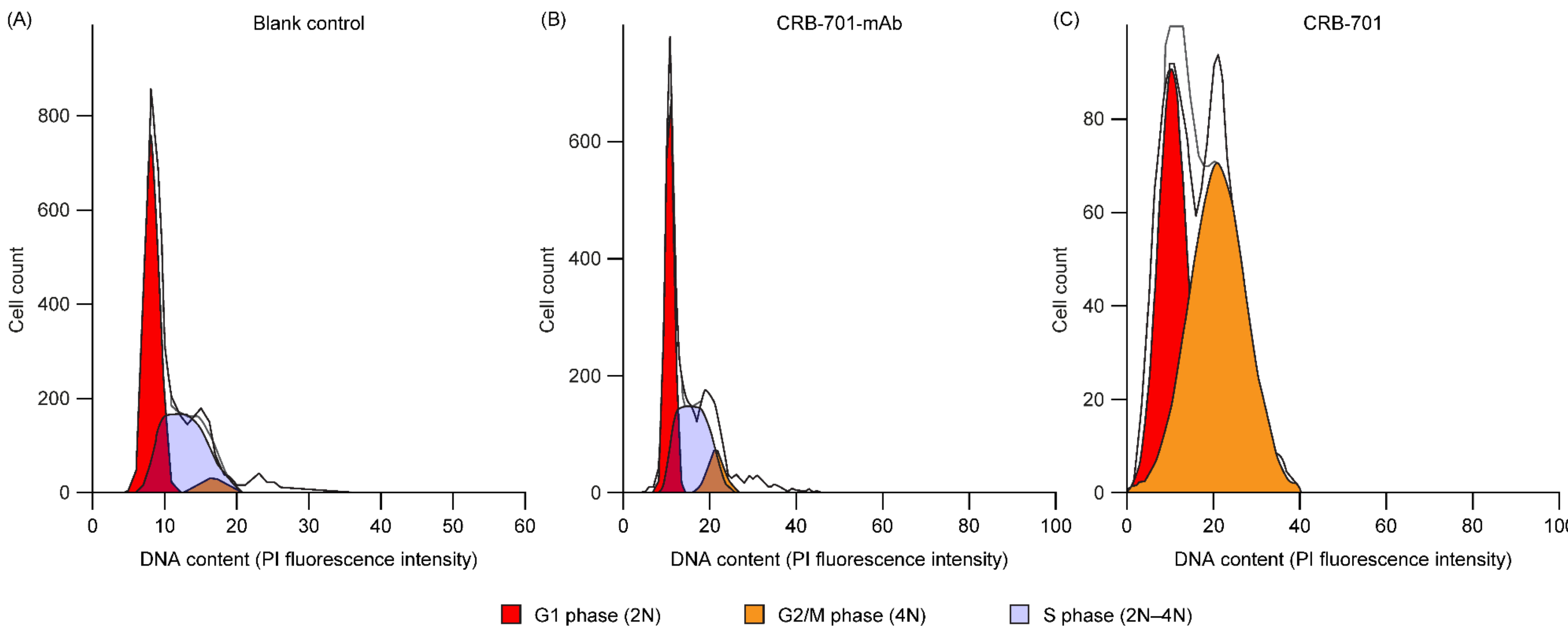

Figure S6. Cell cycle analysis of 293T-NECTIN4 cells by flow cytometry using PI staining. Cells were treated with (A) blank control (cell culture medium), (B) 10 µg/mL CRB-701-mAb or (C) 10 µg/mL CRB-701 for 66 ± 3 hours. Fluorescence intensity histograms were fitted to a cell cycle distribution model based on chromosomal content: G1 phase (2N), S phase (2N–4N), and G2/M phase (4N).

mAb, monoclonal antibody; PI, propidium iodide.
