# Supplementary material for: CRB-701: A Second-generation Nectin-4–Targeted Antibody–Drug Conjugate with Optimized Stability and Pharmacokinetics for the Treatment of Solid Tumors
Source: Cancer Res Commun. 2026 Sep 15;6(9):2164–76. doi: 10.1158/2767-9764.CRC-25-0799 (PMC13576400; doi:10.1158/2767-9764.CRC-25-0799)
Supplement: Supplementary file 8 [file crc-25-0799_figure_s7_suppsf7.pdf]

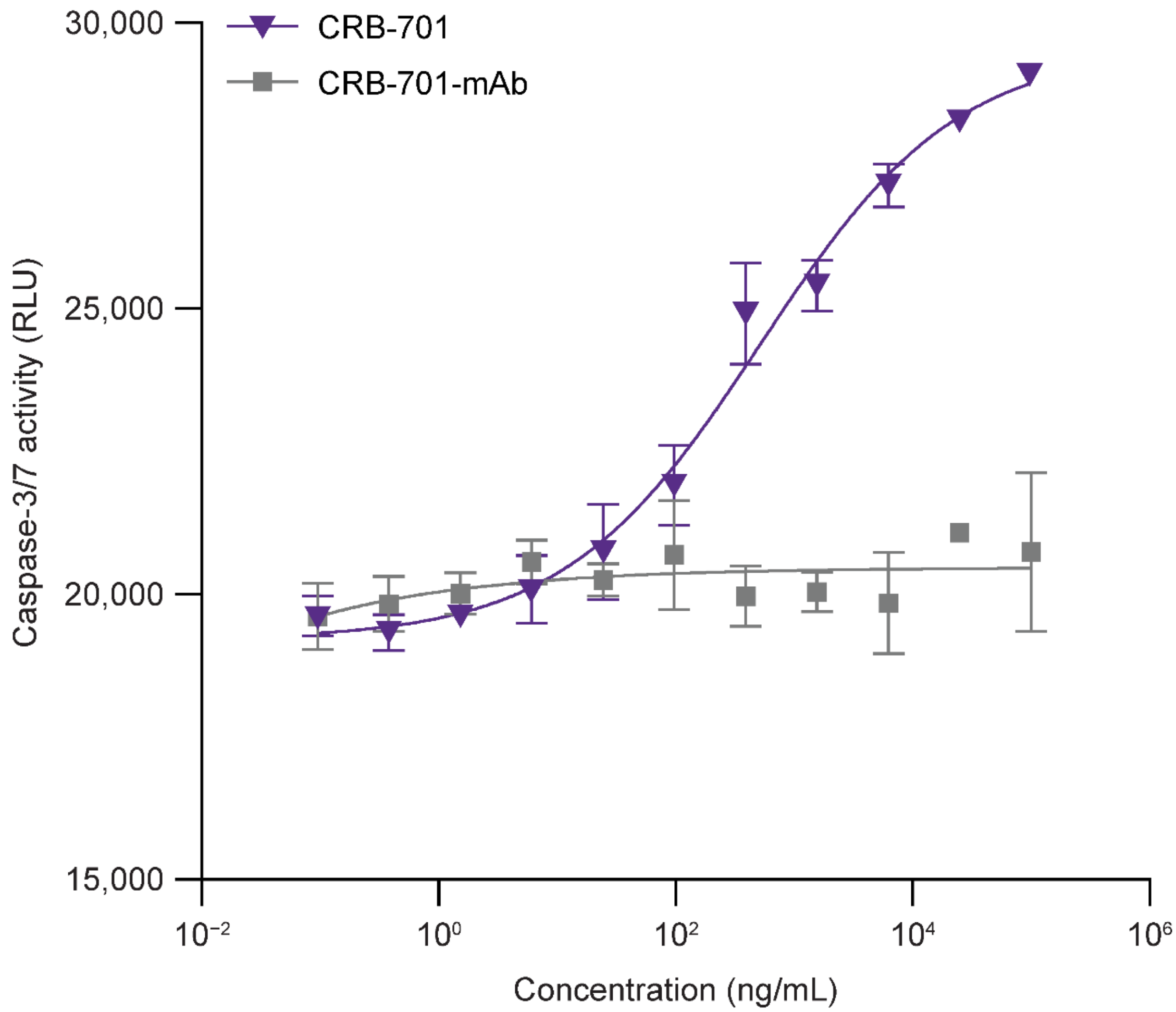

Figure S7. Effect of CRB-701 and CRB-701-mAb on caspase-3/7 activity in PC-3-NECTIN4 cells measured with a Caspase-Glo 3/7 Assay Kit. Cells were treated with CRB-701 or CRB-701-mAb for 24 ±3 hours. Data are presented as mean ±SD from three technical replicates at each concentration; data were fit to a four-parameter model.

mAb, monoclonal antibody; RLU, relative light unit; SD, standard deviation.
