# Supplementary material for: CRB-701: A Second-generation Nectin-4–Targeted Antibody–Drug Conjugate with Optimized Stability and Pharmacokinetics for the Treatment of Solid Tumors
Source: Cancer Res Commun. 2026 Sep 15;6(9):2164–76. doi: 10.1158/2767-9764.CRC-25-0799 (PMC13576400; doi:10.1158/2767-9764.CRC-25-0799)
Supplement: Supplementary file 9 [file crc-25-0799_figure_s8_suppsf8.pdf]

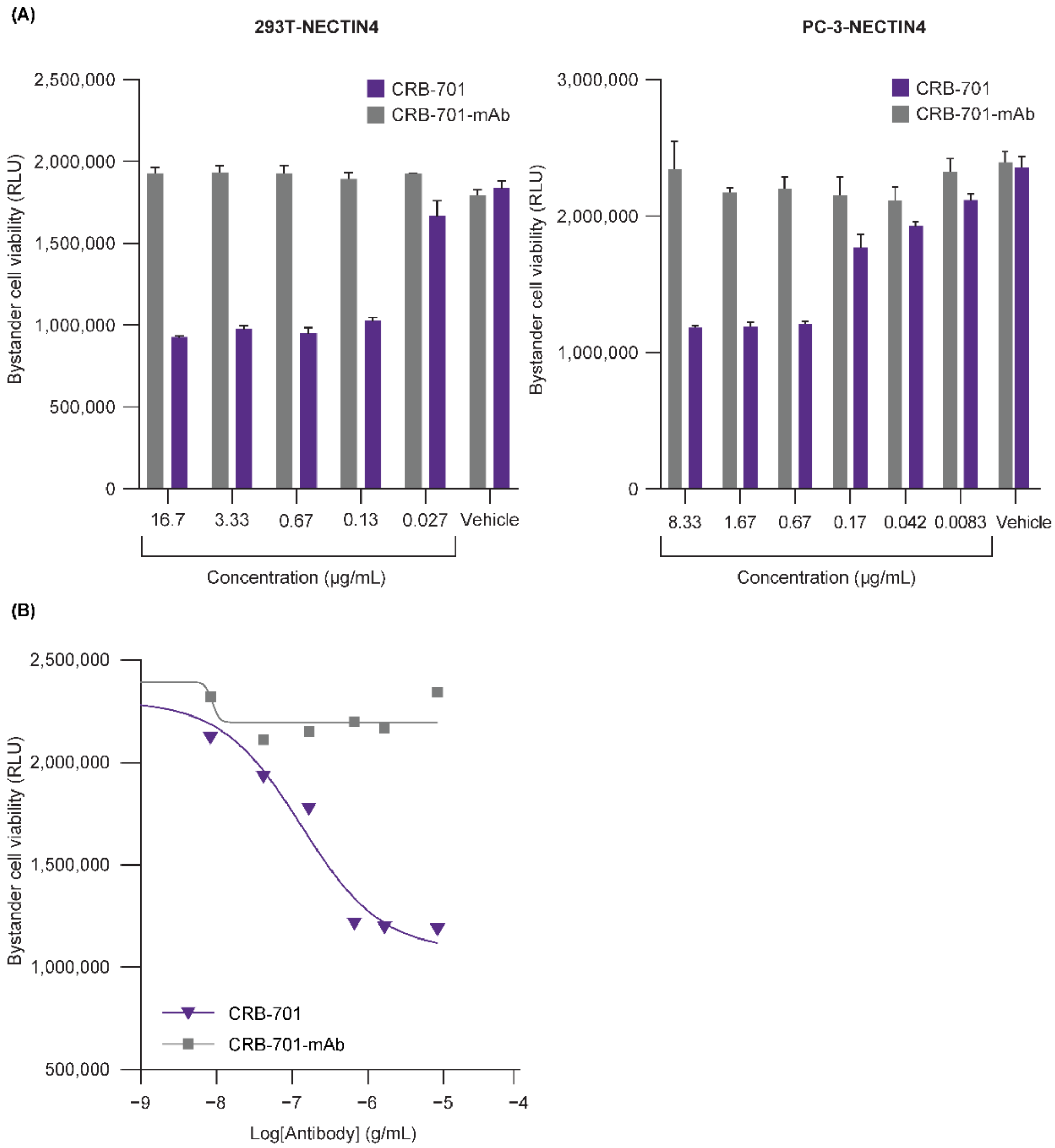

Figure S8. (A) Bystander-mediated cytotoxicity of CRB-701 or CRB-701-mAb in 293T-NECTIN4 and PC-3-NECTIN4 cells as determined by luciferase assay. A reduction in luminescence indicates a reduction in the viability of cocultured HEK293-LUC cells. Data are presented as mean ±SDs of three technical replicates. (B) Dose-response analysis of CRB-701 and CRB-701-mAb bystander-mediated cytotoxicity in PC-3-NECTIN4 cells. Data were fitted using a four-parameter sigmoidal model, yielding an IC_{50} of 1.3×10^{-7} g/mL (130 ng/mL). No cytotoxicity was observed in parental 293T cells at up to 833 ng/mL CRB-701 (**Fig. S4**).

IC_{50}, concentration required to produce 50% maximal inhibition; mAb, monoclonal antibody; RLU, relative light unit; SD, standard deviation.
