# Supplementary material for: CRB-701: A Second-generation Nectin-4–Targeted Antibody–Drug Conjugate with Optimized Stability and Pharmacokinetics for the Treatment of Solid Tumors
Source: Cancer Res Commun. 2026 Sep 15;6(9):2164–76. doi: 10.1158/2767-9764.CRC-25-0799 (PMC13576400; doi:10.1158/2767-9764.CRC-25-0799)
Supplement: Supplementary file 10 [file crc-25-0799_figure_s9_suppsf9.pdf]

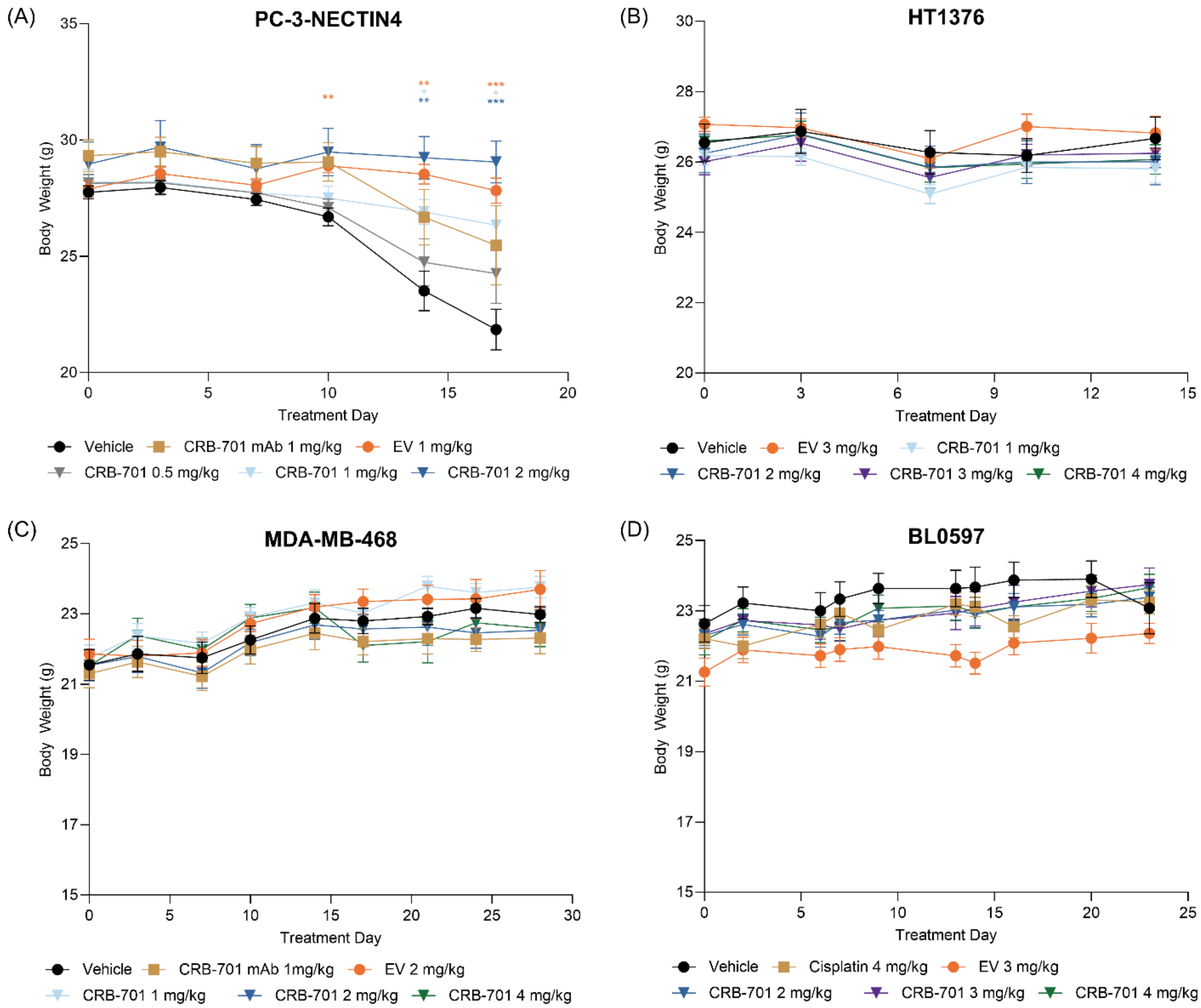

Figure S9. Body weight changes by treatment groups in four xenograft mouse models. (A) PC-3-NECTIN4 human prostate cancer CDX model (n = 7 per group). (B) HT1376 human bladder cancer CDX model (n = 8 per group). (C) MDA-MB-468 human triple-negative breast cancer CDX model (n = 8 per group). (D) BL0597 human bladder cancer PDX model (n = 8 per group). Treatments were administered as a single intravenous dose on day 0 in CDX models. In the PDX model, treatments were administered once a week for three weeks. Body weights were recorded twice a week. Data are presented as mean ±SEM. *$p < 0.05$, **$p > 0.01$; ***$p < 0.001$ versus vehicle.

CDX, cell line-derived xenograft; EV, enfortumab vedotin; mAb, monoclonal antibody; PDX, patient-derived xenograft; SEM, standard error of the mean.
