# Supplementary material for: CRB-701: A Second-generation Nectin-4–Targeted Antibody–Drug Conjugate with Optimized Stability and Pharmacokinetics for the Treatment of Solid Tumors
Source: Cancer Res Commun. 2026 Sep 15;6(9):2164–76. doi: 10.1158/2767-9764.CRC-25-0799 (PMC13576400; doi:10.1158/2767-9764.CRC-25-0799)
Supplement: Supplementary file 11 [file crc-25-0799_supplementary_methods_suppsm.pdf]

CRB-701: a Second-Generation Nectin-4 Targeted Antibody–Drug Conjugate with Optimized Stability and Pharmacokinetics for the Treatment of Solid Tumors

Zhaopeng Sun[1], Mo Dan[1], Lu Lv[1], Mingyue Shen[1], Can Yuan[1], Congcong Niu[1], Yang Zhang[1], Xixin Hu[1], Xiwu Hui[1], and Andrew Kolodziej[2]

[1]CSPC Megalith Biopharmaceutical Co., Ltd. Shijiazhuang, China.

[2]Corbus Pharmaceuticals Inc., Norwood, Massachusetts, USA.

Supplementary Methods

Materials for protein and antibody-based assays

Recombinant human nectin-1 (Cat. # 11611-H08H), nectin-2 (Cat. # 10005-H08H), nectin-3 (Cat. # 10852-H08H), and nectin-4 (Cat. # 19771-H08H) proteins with histidine tags were obtained from Sino Biological. Cynomolgus monkey (Cat. # NE4-C52H4) and mouse (Cat. # NE4-M52H3) histidine-tagged nectin-4 proteins were sourced from ACRO BioSystems, while rat nectin-4 with a histidine tag was obtained from R&D Systems (Cat. # 9997-N4-050). HRP-conjugated goat anti-human IgG $F(ab')_2$ secondary antibody was purchased from Jackson ImmunoResearch (Cat. # 109-036-098). Alexa Fluor 488 conjugated goat anti-human IgG (H+L) cross-adsorbed secondary antibody was obtained from Invitrogen (Cat. # A11013). EV was provided by Seattle Genetics, Inc. Cisplatin was sourced from Qilu Pharmaceutical (Hainan) Co., Ltd. Mouse anti-MMAE antibody (IgG1) was obtained from ACRO BioSystems. HRP-conjugated goat anti-human IgG (heavy and light chain) monkey-adsorbed antibody was purchased from Bethyl Laboratories. MMAE and D8-MMAE were supplied by MedChemExpress. Tetramethylbenzidine (TMB) substrate was obtained from Solarbio (Cat. # PR1210).

Nectin selectivity

His-tagged human nectin-1, nectin-2, nectin-3, nectin-4, and PVR proteins were used as coating antigens in 96-well microtiter plates, diluted in carbonate buffer, pH 9.4 (Thermo

Scientific TL276107) at 0.5 μg/mL (100 μL/well), and incubated overnight at 4°C. The plates were further blocked with 1% HAS in PBS for 2 hours at room temperature, and washed three times. A sixfold dilution series of CRB-701 from 5 μg/mL to 0.083 μg/mL (11 concentration levels) was added to each plate in triplicate, along with a buffer control. Dilutions were added at 100 mL/well, and shaken at room temperature for 2 hours. The plate was washed four times with PBS containing 0.1% Tween-20 (PBST), and Goat Anti-Human IgG F(ab)'2-HRP was added at a 1:5000 dilution. The plate was shaken at room temperature for 1 hour and washed four times with PBST and developed with TMB substrate solutions. Absorbance was read at OD45/650 nm and data (absorbance vs concentration) were fit to a four-parameter dose response curve to determine the half-maximum binding concentration (ED_{50}).

Fc receptors and C1q protein binding affinity assays

Binding affinity of CRB-701/CRB-701-mAb to human Fc receptors and human C1q protein was assessed via BLI using an Octet RED96e System (Pall FortéBio), following a similar protocol as described in the human nectin-4 binding affinity assays.

FcRn (Cat. # 11604-H27H), FcγRIa (Cat. # 10256-H08H), FcγRIIa (Cat. # 10374-H08H1), FcγRIIb (Cat. # 10257-H08H1), FcγRIIIa F176 (Cat. # 10389-H08H), and FcγRIIIa V176 (Cat. # 10389-H08H1) proteins (Sino Biological) were immobilized onto HIS1K biosensor chips (Sartorius, Cat. # 18-5120); human C1q protein (Abcam, Cat. # ab278067) was immobilized onto a Protein L BioSensor (Sartorius, Cat. # 18-5085). Binding activities of seven concentrations of CRB-701 or CRB-701-mAb were tested with twofold serial dilutions starting from 500 nM (FcRN), 100 nM (FcγRIa), 5000 nM (FcγRIIa, FcγRIIb and FcγRIIIa V176), or 800 nM (C1q); binding activities of five concentrations of CRB-701 or CRB-701-mAb were tested with twofold serial dilutions starting from 5000 nM for FcγRIIIa F176.

***In vitro* stability and MMAE release**

The *in vitro* stability of CRB-701 was evaluated in Sprague Dawley rat, cynomolgus monkey, and human plasma or PBST as a protein- and enzyme-free control. CRB-701 was tested at concentrations of 2 µM (rat plasma), 0.2 µM (monkey plasma), and 0.2 µM

(human plasma). CRB-701 was incubated in plasma samples or PBST at 37 °C for up to 336 hours. PBST was included to assess nonenzymatic degradation and to confirm the reliability of the assay system.

Aliquots were collected at 0, 24, 48, 72, 96, 168, and 336 hours. All conditions were tested in duplicate. For each timepoint, released MMAE was quantified using a validated LC-MS/MS method as described below. Concentrations of intact CRB-701 and tAb were determined using validated ELISA-based assays as described below.

The degradation half-life ($T_{1/2}$) was calculated using the equation: $T_{1/2} = 0.693/k$, in which k is the negative slope of the natural logarithm of the percentage of drug remaining versus time. For each incubation condition, the following metrics were calculated: MMAE production (%) = (measured MMAE concentration / theoretical molarity) × 100; ADC remaining (%) = (measured ADC concentration at time t / ADC concentration at time 0) × 100 and tAb remaining (%) = (measured tAb concentration at time t / tAb concentration at time 0) × 100.

Quantification of ADC

CRB-701 ADC concentrations were measured using a validated sandwich ELISA. Mouse anti-MMAE IgG1 antibody (1 µg/mL in DPBS) was immobilized onto wells of a 96-well plate. Following a 2-hour incubation with diluted serum or tissue homogenate samples, plates were washed and incubated with HRP-conjugated goat anti-human IgG (heavy and light chain) monkey-adsorbed antibody (1:40,000 dilution in PBST containing 1% bovine serum albumin) for 1 hour. Detection was performed using TMB substrate, and the reaction was quenched with 2 M sulfuric acid. Absorbance was read at 450/630 nm using a microplate reader. CRB-701 concentrations were calculated against a standard curve (calibration range: 16.0–512 ng/mL; minimum required dilution [MRD]: 1:20).

Quantification of tAb

CRB-701 tAb levels were measured using a validated indirect ELISA. Recombinant His-tagged human nectin-4 protein (1 µg/mL in DPBS) was immobilized onto wells of a 96-well plate. Following a 2-hour incubation with diluted serum or tissue homogenate

samples, plates were washed and incubated with HRP-conjugated goat $F(ab')_2$ anti-human IgG secondary antibody (1:40,000 dilution in DPBS with Tween 20 and 1% BSA) for 1 hour. Detection was performed using TMB substrate, and the reaction was quenched with 2 M sulfuric acid. Absorbance was measured at 450/630 nm. Concentrations were calculated against standard curves with the following calibration ranges: 1.25–40 ng/mL (rat, MRD 1:15) and 6.25–200 ng/mL (cynomolgus monkey, MRD 1:15).

Quantification of MMAE

MMAE concentrations were determined using a validated LC-MS/MS method. Samples were precipitated with acetonitrile and centrifuged at 3200 × g for 10 minutes. For rat plasma, supernatants were further purified using solid-phase extraction 96-well plates (30 mg/2 mL/well). Chromatographic separation was performed using the following columns: mouse serum, Acquity UPLC BEH C18 (2.1 × 50 mm, 1.7 µm); mouse tumor, Acquity UPLC HSS T3 (2.1 × 100 mm, 1.8 µm); rat plasma, Ultimate XB-C18 (2.1 × 50 mm, 5 µm); cynomolgus monkey plasma, Acquity UPLC HSS T3 (2.1 × 100 mm, 1.8 µm). Elution was achieved using a gradient of acetonitrile and formic acid. MMAE was detected on a QTRAP 6500 mass spectrometer (Applied Biosystems) using multiple reaction monitoring with the following transitions: mouse serum, m/z 718.45 → 152.09; rat serum and mouse tumor, m/z 718.45 → 686.52. MMAE-d_8 was used as an internal standard. Calibration ranges were: rat, 10 pg/mL to 5 ng/mL; cynomolgus monkey, 20 pg/mL to 10 ng/mL; mouse serum, 20 pg/mL to 10 ng/mL; mouse tumor, 40 pg/mL to 20 ng/mL.

Statistical analysis for PDX models

For PDX models, tumor volume comparisons between treatment groups at prespecified time points were conducted using a stepwise approach based on the distribution and variance characteristics of the data.

First, Bartlett's test was used to assess the homogeneity of variances across groups. If the test indicated equal variances ($p \geq 0.05$), one-way ANOVA was performed to evaluate overall differences in group means. When the ANOVA result was statistically

significant ($p < 0.05$), *post hoc* analyses were conducted using Tukey's Honest Significant Difference test for all pairwise comparisons and Dunnett's test for comparisons between each treatment group and the vehicle control.

If Bartlett's test indicated unequal variances ($p < 0.05$), the nonparametric Kruskal–Wallis test was used to assess differences in group medians. When significant ($p < 0.05$), *post hoc* comparisons were performed using Conover's nonparametric test, either for all pairwise comparisons or for comparisons against the vehicle group, with single-step *p*-value adjustment.

In addition to the above, exploratory pairwise comparisons were conducted without correction for multiple testing. For each pair of groups, Bartlett's test was used to assess variance homogeneity. If variances were unequal ($p \geq 0.05$), Welch's t-test was applied; otherwise, the Mann–Whitney U test was used. Nominal (uncorrected) *p*-values from these tests were reported.

All statistical analyses were performed using R software (version 3.3.1). All tests were two-sided unless otherwise specified, and *p*-values < 0.05 were considered statistically significant.
