# Supplementary material for: CRB-701: A Second-generation Nectin-4–Targeted Antibody–Drug Conjugate with Optimized Stability and Pharmacokinetics for the Treatment of Solid Tumors
Source: Cancer Res Commun. 2026 Sep 15;6(9):2164–76. doi: 10.1158/2767-9764.CRC-25-0799 (PMC13576400; doi:10.1158/2767-9764.CRC-25-0799)
Supplement: Supplementary file 12 [file crc-25-0799_table_s10_suppst10.pdf]

Table S10. Mean toxicokinetic parameters at day 1 and day 22 for CRB-701 and EV analytes (ADC, tAb, and MMAE) following four weekly doses of 6 mg/kg CRB-701 or EV in cynomolgus monkeys

	**CRB-701**		**EV**	
**Parameter**	**Day 1**	**Day 22**	**Day 1**	**Day 22**
**Serum ADC**				
C_{max} (µg/mL)	242.66	174.99	161.34	137.51
AUC_{last} (h*µg/mL)	9894.23	12,755.92	8139.21	7114.77
AUC_{inf} (h*µg/mL)	15524.44	19,459.54	9341.03	7982.08
$T_{1/2}$ (hours)	118.98	114.69	57.35	51.31
R_{acc}	-	1.29	-	0.87
**Serum tAb**				
C_{max} (µg/mL)	221.87	177.93	149.80	162.27
AUC_{last} (h*µg/mL)	9760.18	13,024.27	8598.93	9512.88
AUC_{inf} (h*µg/mL)	14,258.31	20,049.66	10,955.19	11,820.36
$T_{1/2}$ (hours)	101.20	116.18	77.79	72.66
R_{acc}	-	1.33	-	1.11
**Plasma MMAE**				
C_{max} (pg/mL)	92.34	237.61	199.45	441.26
AUC_{last} (h*pg/mL)	10,358.36	28,531.20	25,459.89	49,262.37
T_{max} (hours)[a]	72 (48–96)	36 (24–48)	72 (48–96)	36 (24–48)
R_{acc}	-	2.75	-	1.93

[a]Median (range).

Data shown are mean values from one female and one male cynomolgus monkey; T_{last} was 168 hours.

ADC, antibody–drug conjugate; AUC_{inf}, area under the time–concentration curve from time 0 to infinity; AUC_{last}, area under the time–concentration curve from dosing to the time of the last measured concentration; maximum concentration; C_{max}, maximum concentration; EV, enfortumab vedotin; MMAE, monomethyl auristatin E; $R_{acc,}$

accumulation ratio; $T_{1/2}$, half-life; tAb, total antibody; T_{last}, time of last measurable concentration; T_{max}, time to reach C_{max}.
