# Supplementary material for: CRB-701: A Second-generation Nectin-4–Targeted Antibody–Drug Conjugate with Optimized Stability and Pharmacokinetics for the Treatment of Solid Tumors
Source: Cancer Res Commun. 2026 Sep 15;6(9):2164–76. doi: 10.1158/2767-9764.CRC-25-0799 (PMC13576400; doi:10.1158/2767-9764.CRC-25-0799)
Supplement: Supplementary file 13 [file crc-25-0799_table_s11_suppst11.pdf]

Table S11. Serum and tumor concentrations of CRB-701 and MMAE over time in the MDA-MB-468 human triple-negative breast cancer CDX model following a single 6 mg/kg dose of CRB-701.

	**Mean (SD) concentration over time (hours)**				
	**0.5 h**	**24 h**	**96 h**	**168 h**	**384 h**
**CRB-701**					
Serum, µg/mL	76.4 (14.50)	34.0 (2.80)	2.53 (3.29)	0.14 (0.14)	0.01 (0.00)
Tumor, µg/g	1.82 (0.46)	8.29 (0.94)	2.86 (0.58)	5.42 (7.55)	0.75[a]
**MMAE**					
Serum, ng/mL	2.68 (0.40)	0.88 (0.26)	0.39 (0.26)	0.05 (0.04)	0.03 (0.01)
Tumor, ng/g	2.93 (0.12)	58.5 (4.67)	88.7 (6.01)	55.7 (26.10)	11.8 (18.20)

[a]SD was not calculated because measurements for two of the three mice were below the lower limit of quantification.

Data are mean (SD) from three mice per time point.

CDX, cell line-derived xenograft; MMAE, monomethyl auristatin E; SD, standard deviation.
