# Supplementary material for: CRB-701: A Second-generation Nectin-4–Targeted Antibody–Drug Conjugate with Optimized Stability and Pharmacokinetics for the Treatment of Solid Tumors
Source: Cancer Res Commun. 2026 Sep 15;6(9):2164–76. doi: 10.1158/2767-9764.CRC-25-0799 (PMC13576400; doi:10.1158/2767-9764.CRC-25-0799)
Supplement: Supplementary file 14 [file crc-25-0799_table_s12_suppst12.pdf]

Table S12. Skin toxicity events in cynomolgus monkey

**Sex**	Male				Female			
**Group number and treatment dose**	Group 1 0 mg/kg	Group 2 2.5 mg/kg	Group 3 7.5 mg/kg	Group 4 15 mg/kg	Group 1 0 mg/kg	Group 2 2.5 mg/kg	Group 3 7.5 mg/kg	Group 4 15 mg/kg
**Animals in group, n**	3	3	3	3	3	3	3	3
**Animals examined, n**	0	0	3	3	0	0	3	3
**Skin, n**								
Toxicity event<br>Severity								
Hyperplasia; conjunctiva; epithelium	–	–	0	0	–	–	0	0
Hyperplasia; epidermal	–	–	2	2	–	–	2	1
Minimal	–	–	1	1	–	–	0	0
Slight	–	–	1	0	–	–	2	1
Moderate	–	–	0	1	–	–	0	0
Infiltrate; neutrophilic, conjunctiva	–	–	0	2	–	–	0	1
Minimal	–	–	0	0	–	–	0	1
Slight	–	–	0	2	–	–	0	0
Inflammation; neutrophilic, conjunctiva	–	–	0	0	–	–	0	0
Inflammation; neutrophilic, dermis; subcutis	–	–	0	1	–	–	0	0
Moderate	–	–	0	1	–	–	0	0
Inflammation; neutrophilic, epidermal; dermis	–	–	0	0	–	–	0	0
Mitotic figure; increased, epithelium, conjunctiva	–	–	1	2	–	–	0	0
Minimal	–	–	1	1	–	–	0	0
Slight	–	–	0	1	–	–	0	0
Mitotic figure; increased, epithelium, conjunctiva; adnexal	–	–	0	0	–	–	0	1
Slight	–	–	0	0	–	–	0	1
Mitotic figure; increased, epithelium; conjunctiva	–	–	0	0	–	–	0	0
Mitotic figure; increased, epidermal	–	–	2	2	–	–	1	1
Minimal	–	–	1	0	–	–	0	1

Slight	–	–	1	1	–	–	1	0
Moderate	–	–	0	1	–	–	0	0
Mitotic figure; increased, epithelium, epidermal	–	–	0	0	–	–	0	0
Mitotic figure; increased, epidermal; adnexal	–	–	1	1	–	–	2	1
Minimal	–	–	0	0	–	–	0	0
Slight	–	–	1	1	–	–	2	1
Apoptosis; increased, epithelium, conjunctiva	–	–	0	1	–	–	0	0
Minimal	–	–	0	1	–	–	0	0
Apoptosis; increased, conjunctiva; epithelium	–	–	1	0	–	–	0	1
Minimal	–	–	1	0	–	–	0	1
Apoptosis; increased, epidermal	–	–	3	2	–	–	2	2
Minimal	–	–	2	2	–	–	2	1
Slight	–	–	1	0	–	–	0	1
Crust, epidermal	–	–	0	1	–	–	0	0
Moderate	–	–	0	1	–	–	0	0
Edema; muscularis	–	–	0	0	–	–	0	0
Ulcer	–	–	0	0	–	–	0	0

**Sex**	Male				Female			
**Group number and treatment dose**	Group 1 0 mg/kg	Group 2 2.5 mg/kg	Group 3 7.5mg/kg	Group 4 15 mg/kg	Group 1 0 mg/kg	Group 2 2.5 mg/kg	Group 3 7.5mg/kg	Group 4 15 mg/kg
**Animals in group, n**	3	3	3	3	3	3	3	3
**Animals examined, n**	3	3	3	3	3	3	3	3
**Skin, mammary, n**								
Toxicity event<br>Severity								
**Normal**	3	2	0	0	3	1	0	0
**Mitotic figure; increase, epidermal**	0	1	2	3	0	1	2	3
Minimal	0	1	2	1	0	1	2	2

Slight	0	0	0	2	0	0	0	1
**Mitotic figure; increase, epidermal; adnexal**	0	0	1	0	0	1	1	0
Minimal	0	0	0	0	0	1	0	0
Slight	0	0	1	0	0	0	1	0
**Apoptosis; increased, epidermal**	0	0	3	2	0	0	1	2
Minimal	0	0	3	1	0	0	1	2
Slight	0	0	0	1	0	0	0	0
**Hyperplasia; epidermal**	0	0	1	1	0	0	0	0
Minimal	0	0	1	1	0	0	0	0
