# Supplementary material for: CRB-701: A Second-generation Nectin-4–Targeted Antibody–Drug Conjugate with Optimized Stability and Pharmacokinetics for the Treatment of Solid Tumors
Source: Cancer Res Commun. 2026 Sep 15;6(9):2164–76. doi: 10.1158/2767-9764.CRC-25-0799 (PMC13576400; doi:10.1158/2767-9764.CRC-25-0799)
Supplement: Supplementary file 15 [file crc-25-0799_table_s13_suppst13.pdf]

Table S13. Toxicokinetic exposures from 4-week repeat dose GLP toxicology studies in the rat and in cynomolgus monkeys following single and repeat doses of CRB-701 on Day 1 and Day 29 and EV on Day 1 and Day 22.

Species	Day	Drug	Dose	C_{max} (µg/mL)	AUC_{last} (h*µg /mL)	C_{ave} (µg/h/mL)
Rat	1	CRB-701	5	87.1	3858.4	11.5
			15	278.6	14,469.5	43.1
			30	557.4	33,354.3	99.3
	1	EV	2	57.3	1150	6.8
			5	131	1630	9.7
			10	276	6030	35.9
	29	CRB-701	5	93.2	1833.5	5.5
			15	373.5	19,303.6	57.5
			30	724.9	55,749.1	165.9
	22	EV	2	54.1	862	5.1
			5	111	1640	9.8
			10	206	3920	23.3
Monkey	1	CRB-701	2.5	54.1	3494.1	10.4
			7.5	171.0	13,960.7	41.6
			15	317.2	29,568.4	88.0
	1	EV	1	24.6	631	3.8
			3	76.6	2430	14.5
			6	151	5050	30.1
	29	CRB-701	2.5	93.2	1833.5	5.5
			7.5	373.5	19,303.6	57.5
			15	724.9	55,749.1	165.9
	22	EV	1	21.2	466	2.8
			3	63.7	856	5.1
			6	137	6310	37.6

Toxicokinetic parameters (C_{max}, AUC_{last}, and C_{ave}). C_{ave} was calculated by dividing AUC_{last} by the dosing interval, 336 h for CRB-701 and 168 h for EV.
