# Supplementary material for: CRB-701: A Second-generation Nectin-4–Targeted Antibody–Drug Conjugate with Optimized Stability and Pharmacokinetics for the Treatment of Solid Tumors
Source: Cancer Res Commun. 2026 Sep 15;6(9):2164–76. doi: 10.1158/2767-9764.CRC-25-0799 (PMC13576400; doi:10.1158/2767-9764.CRC-25-0799)
Supplement: Supplementary file 16 [file crc-25-0799_table_s1_suppst1.pdf]

Table S1. List of key materials, suppliers, and (where applicable) RRIDs

Cell lines

**Name**	**Supplier**	**Catalogue number**	**RRID (if available)**
PC-3 prostate carcinoma cell line	CoBioer	CBP61712	CVCL_0035
PC-3-NECTIN4 (PC3-PVRL4) cell line	Kyinno Biotechnology	KC-1794-DW	NA
293T renal cell line	Kyinno Biotechnology	NA	CVCL_0063
293T-NECTIN4	Kyinno Biotechnology	KC-1523	CVCL_E8M6
HEK293-LUC	Shanghai MeiXuan Biological	NA	CVCL_C9C7
T47D breast cancer cell line	American Type Culture Collection	HTB-133	CVCL_0553
SK-BR-3 breast cancer cell line	American Type Culture Collection	HTB-30	CVCL_0033
MDA-MB-468 breast cancer cell line	American Type Culture Collection	HTB-132	CVCL_0419
Jurkat/hFcγRIIIa-NFAT cells	National Institutes for Food and Drug Control (China)	NA	NA
Chinese Hamster Ovary cell line (CHOK1SV GS-KO)	Lonza	NA	CVCL_DR97
HT-1376 bladder carcinoma cell	American Type Culture Collection	CRL-1472	CVCL_1292

Animals

**Name**	**Supplier**	**Catalogue number**	**RRID (if available)**
BALB/c mice	Beijing Anikeeper Biotech Co., Ltd	NA	MGI:2161072
BALB/c nude mice	Beijing Anikeeper Biotech Co., Ltd	NA	IMSR_CRL:194
NU/NU mice	Vital River Laboratory Animal Technology Co., Ltd	403	IMSR_CRL:088
NOD/SCID mice	Vital River Laboratory Animal Technology Co., Ltd	406	IMSR_CRL:394
Sprague Dawley rat	Vital River Laboratory Animal Technology Co., Ltd	001	RGD_734476
Cynomolgus monkey	Conghua Huazhen Animal Farm	NA	NA

Antibodies and proteins

**Name**	**Supplier**	**Catalogue number**	**RRID (if available)**
HRP-conjugated goat anti-human IgG ($F(ab')_2$)	Jackson ImmunoResearch	109-036-098	AB_2337596

Alexa Fluor 488 conjugated goat anti-human IgG (H+L)	Invitrogen	A11013	AB_2534080
Mouse anti-MMAE antibody (IgG1)	ACRO BioSystems	MME-M5252	NA
HRP-conjugated goat anti-human IgG (heavy and light chain) monkey-adsorbed antibody	Bethyl Laboratories	NA	NA
Histidine tagged recombinant human nectin-1	Sino Biological	11611-H08H	NA
Histidine tagged recombinant human nectin-2	Sino Biological	10005-H08H	NA
Histidine tagged recombinant human nectin-3	Sino Biological	10852-H08H	NA
Histidine tagged recombinant human nectin-4	Sino Biological	19771-H08H	NA
Histidine tagged mouse nectin-4	ACRO BioSystems	NE4-M52H3	NA
Histidine tagged cynomolgus monkey nectin-4	ACRO BioSystems	NE4-C52H4	NA
Histidine tagged rat nectin-4	R&D Systems	9997-N4-050	NA
FcRn protein	Sino Biological	11604-H27H	NA
FcγRIa protein	Sino Biological	10256-H08H	NA
FcγRIIa protein	Sino Biological	10374-H08H1	NA
FcγRIIb protein	Sino Biological	10259-H08H1	NA
FcγRIIIa F176 protein	Sino Biological	10389-H08H	NA
FcγRIIIa V176 protein	Sino Biological	10389-H08H1	NA
Human C1q protein	Abcam	ab278067	NA
PADCEV (enfortumab vedotin)	Seattle Genetics	Lot 107386	NA

Software

Name	Supplier	Catalogue number	RRID (if available)
GraphPad Prism	GraphPad	NA	SCR_002798
Phoenix WinNonlin version 8.0	Certara	NA	SCR_024504

Hardware

Name	Supplier	Catalogue number	RRID (if available)
Attune NxT Flow Cytometer	Thermo Fisher	NA	SCR_019590
Octet RED96e System	Pall FortéBio	NA	NA
AB SCIEX LC-30 system	Sciex	NA	NA
AB SCIEX Triple Quad 5500 mass spectrometer	Sciex	NA	NA

NA, not applicable; RRID, Research Resource Identification.
