# Supplementary material for: CRB-701: A Second-generation Nectin-4–Targeted Antibody–Drug Conjugate with Optimized Stability and Pharmacokinetics for the Treatment of Solid Tumors
Source: Cancer Res Commun. 2026 Sep 15;6(9):2164–76. doi: 10.1158/2767-9764.CRC-25-0799 (PMC13576400; doi:10.1158/2767-9764.CRC-25-0799)
Supplement: Supplementary file 17 [file crc-25-0799_table_s2_suppst2.pdf]

Table S2. Intracellular MMAE release in PC-3 and PC-3-NECTIN4 cells following treatment with 2 μg/mL CRB-701

	Released MMAE (ng/mL)
**PC-3**	
0 hours	BLQ
6 hours	BLQ
24 hours	BLQ
**PC-3-NECTIN4**	
0 hours	BLQ
6 hours	0.21
24 hours	0.55

MMAE release was determined by LC-MS/MS. Lower limit of quantification: 0.023 ng/mL.

BLQ, below limit of quantification; LC-MS/MS, liquid chromatography–tandem mass spectrometry; MMAE, monomethyl auristatin E.
