# Supplementary material for: CRB-701: A Second-generation Nectin-4–Targeted Antibody–Drug Conjugate with Optimized Stability and Pharmacokinetics for the Treatment of Solid Tumors
Source: Cancer Res Commun. 2026 Sep 15;6(9):2164–76. doi: 10.1158/2767-9764.CRC-25-0799 (PMC13576400; doi:10.1158/2767-9764.CRC-25-0799)
Supplement: Supplementary file 18 [file crc-25-0799_table_s3_suppst3.pdf]

Table S3. Effect of CRB-701-mAb and CRB-701 on 293T-NECTIN4 cell cycle distribution

Treatment	Cells in G1 phase (%)	Cells in S phase (%)	Cells in G2/M phase (%)
Vehicle	55.67	40.40	3.93
CRB-701-mAb	48.06	42.18	9.76
CRB-701	39.64	0.00	60.36

Cell cycle distribution was determined based on chromosomal content by flow cytometry using PI staining: G1 phase (2N), S phase (2N–4N) and G2/M phase (4N).

mAb, monoclonal antibody; PI, propidium iodide.
