# Supplementary material for: CRB-701: A Second-generation Nectin-4–Targeted Antibody–Drug Conjugate with Optimized Stability and Pharmacokinetics for the Treatment of Solid Tumors
Source: Cancer Res Commun. 2026 Sep 15;6(9):2164–76. doi: 10.1158/2767-9764.CRC-25-0799 (PMC13576400; doi:10.1158/2767-9764.CRC-25-0799)
Supplement: Supplementary file 19 [file crc-25-0799_table_s4_suppst4.pdf]

Table S4. Apoptosis induction in 293T-NECTIN4 cells following treatment with 10 μg/mL CRB-701 or CRB-701-mAb, or vehicle

Treatment	Live cells (%)	Early apoptotic cells (%)	Late apoptotic cells (%)
Vehicle	78.9	6.8	7.2
CRB-701-mAb	72.3	15.9	5.5
CRB-701	29.2	51.1	18.3

The proportion of live cells ($PI^{-}/FITC^{-}$), early-stage apoptotic cells ($PI^{-}/FITC^{+}$), and late-stage apoptotic cells ($PI^{+}/FITC^{+}$) was determined using flow cytometry.

FITC, fluorescein Isothiocyanate; mAb, monoclonal antibody; PI, propidium iodide**.**
