# Supplementary material for: CRB-701: A Second-generation Nectin-4–Targeted Antibody–Drug Conjugate with Optimized Stability and Pharmacokinetics for the Treatment of Solid Tumors
Source: Cancer Res Commun. 2026 Sep 15;6(9):2164–76. doi: 10.1158/2767-9764.CRC-25-0799 (PMC13576400; doi:10.1158/2767-9764.CRC-25-0799)
Supplement: Supplementary file 20 [file crc-25-0799_table_s5_suppst5.pdf]

Table S5. Binding affinities of CRB-701 and CRB-701-mAb to human Fc receptors and C1q determined by BLI

	CRB-701 mAb binding affinity, K_d (M)		CRB-701 binding affinity, K_d (M)	
	**Mean**	**SD**	**Mean**	**SD**
**FcRn**	2.7×10^{-8}	0.29×10^{-8}	3.0×10^{-8}	0.22×10^{-8}
**FcγRIa**	4.1×10^{-9}	0.78×10^{-6}	3.8×10^{-9}	0.69×10^{-6}
**FcγRIIa**	1.4×10^{-6}	0.21×10^{-6}	3.1×10^{-6}	0.53×10^{-6}
**FcγRIIb**	2.9×10^{-6}	0.20×10^{-6}	5.6×10^{-6}	0.46×10^{-6}
**FcγRIIIa F176**	3.8×10^{-6}	0.36×10^{-6}	3.4×10^{-6}	1.41×10^{-6}
**FcγRIIIa V176**	1.1×10^{-6}	0.16×10^{-6}	3.9×10^{-6}	1.18×10^{-6}
**C1q**	6.3×10^{-9}	0.11×10^{-9}	6.5×10^{-9}	0.12×10^{-9}

Mean and SD values are derived from three manufactured lots of CRB-701-mAb and four manufactured lots of CRB-701.

BLI, biolayer interferometry; K_d, binding affinity (k_{off}/k_{on}); mAb, monoclonal antibody; SD, standard deviation.
