# Supplementary material for: CRB-701: A Second-generation Nectin-4–Targeted Antibody–Drug Conjugate with Optimized Stability and Pharmacokinetics for the Treatment of Solid Tumors
Source: Cancer Res Commun. 2026 Sep 15;6(9):2164–76. doi: 10.1158/2767-9764.CRC-25-0799 (PMC13576400; doi:10.1158/2767-9764.CRC-25-0799)
Supplement: Supplementary file 21 [file crc-25-0799_table_s6_suppst6.pdf]

Table S6. Stability of CRB-701 (0.02, 0.2, or 2 μM) over time following incubation with human, cynomolgus monkey, or Sprague Dawley rat plasma, or PBST control

	Remaining CRB-701 (%) over time (hours)						
	**0 h**	**24 h**	**48 h**	**72 h**	**96 h**	**168 h**	**336 h**
**Human**							
CRB-701, 0.02 μM	100	94.96	92.46	88.26	90.27	84.79	79.72
CRB-701, 0.2 μM	100	83.20	81.45	78.91	80.21	79.08	72.99
CRB-701, 2 μM	100	93.38	87.33	82.90	82.93	74.71	70.96
**Cynomolgus monkey**							
CRB-701, 0.02 μM	100	96.57	94.71	88.44	91.42	88.83	75.93
CRB-701, 0.2 μM	100	94.69	89.41	90.54	86.63	85.20	70.30
CRB-701, 2 μM	100	100.74	91.19	95.90	98.90	90.49	75.63
**Sprague Dawley rat**							
CRB-701, 0.02 μM	100	92.95	91.16	86.54	85.74	83.07	73.86
CRB-701, 0.2 μM	100	92.55	86.96	83.50	81.54	82.22	70.99
CRB-701, 2 μM	100	93.42	86.55	82.49	83.96	82.74	73.83
**PBST (control)**							
CRB-701, 0.02 μM	100	95.37	94.20	96.65	106.9	96.98	105.15
CRB-701, 0.2 μM	100	96.90	94.73	97.49	96.76	95.64	103.00
CRB-701, 2 μM	100	100.34	93.12	90.19	92.02	95.25	90.55

CRB-701 levels were determined by ELISA. Remaining CRB-701 levels are shown as the proportion of the total concentration at time point 0.

ELISA, enzyme-linked immunosorbent assay; PBST, phosphate-buffered saline with 0.1% Tween-20.
