# Supplementary material for: CRB-701: A Second-generation Nectin-4–Targeted Antibody–Drug Conjugate with Optimized Stability and Pharmacokinetics for the Treatment of Solid Tumors
Source: Cancer Res Commun. 2026 Sep 15;6(9):2164–76. doi: 10.1158/2767-9764.CRC-25-0799 (PMC13576400; doi:10.1158/2767-9764.CRC-25-0799)
Supplement: Supplementary file 22 [file crc-25-0799_table_s7_suppst7.pdf]

Table S7. Stability of CRB-701-mAb (0.02, 0.2, or 2 μM) over time following incubation with human, cynomolgus monkey, or Sprague Dawley rat plasma, or PBST control

	Remaining CRB-701-mAb (%) over time (hours)						
	**0 h**	**24 h**	**48 h**	**72 h**	**96 h**	**168 h**	**336 h**
**Human**							
CRB-701, 0.02 μM	100	93.45	86.96	87.69	86.15	85.59	73.53
CRB-701, 0.2 μM	100	94.18	90.06	93.26	80.37	84.85	75.53
CRB-701, 2 μM	100	93.42	87.86	83.64	82.05	82.69	66.05
**Cynomolgus monkey**							
CRB-701, 0.02 μM	100	98.69	100.19	98.88	100.29	100.13	89.88
CRB-701, 0.2 μM	100	99.86	98.21	99.53	96.93	94.86	81.08
CRB-701, 2 μM	100	95.40	97.84	100.66	100.06	101.18	88.89
**Sprague Dawley rat**							
CRB-701, 0.02 μM	100	96.77	91.05	90.98	89.48	87.35	81.33
CRB-701, 0.2 μM	100	91.22	88.65	87.60	85.05	83.59	75.91
CRB-701, 2 μM	100	95.47	88.10	88.10	87.13	86.14	80.34
**PBST (control)**							
CRB-701, 0.02 μM	100	98.92	98.46	99.41	160.6	98.11	112.16
CRB-701, 0.2 μM	100	105.36	103.78	103.38	106.91	97.27	115.36
CRB-701, 2 μM	100	103.64	100.99	103.03	101.42	88.00	101.87

CRB-701-mAb levels were determined by ELISA. Remaining CRB-701-mAb levels are shown as the proportion of the total concentration at time point 0. Mean values are shown for two replicates.

ELISA, enzyme-linked immunosorbent assay; mAb, monoclonal antibody; PBST, phosphate-buffered saline with 0.1% Tween-20.
