# Supplementary material for: CRB-701: A Second-generation Nectin-4–Targeted Antibody–Drug Conjugate with Optimized Stability and Pharmacokinetics for the Treatment of Solid Tumors
Source: Cancer Res Commun. 2026 Sep 15;6(9):2164–76. doi: 10.1158/2767-9764.CRC-25-0799 (PMC13576400; doi:10.1158/2767-9764.CRC-25-0799)
Supplement: Supplementary file 23 [file crc-25-0799_table_s8_suppst8.pdf]

Table S8. Mean MMAE release (%) over time following incubation of CRB-701 (0.02, 0.2, or 2 µM) with human, cynomolgus monkey, or Sprague Dawley rat plasma, or PBST control

	**MMAE release (%) over time (hours)**						
	**0 h**	**24 h**	**48 h**	**72 h**	**96 h**	**168 h**	**336 h**
**Human**							
CRB-701, 0.02 µM	0.00	0.00	0.10	0.13	0.21	0.47	0.82
CRB-701, 0.2 µM	0.00	0.05	0.10	0.16	0.22	0.43	0.93
CRB-701, 2 µM	0.00	0.05	0.10	0.17	0.22	0.48	1.00
**Cynomolgus monkey**							
CRB-701, 0.02 µM	0.00	0.00	0.11	0.19	0.24	0.44	0.72
CRB-701, 0.2 µM	0.00	0.06	0.11	0.19	0.26	0.47	0.72
CRB-701, 2 µM	0.00	0.05	0.12	0.19	0.26	0.52	0.75
**Sprague Dawley rat**							
CRB-701, 0.02 µM	0.00	0.06	0.14	0.22	0.29	0.45	0.97
CRB-701, 0.2 µM	0.00	0.07	0.15	0.24	0.30	0.55	1.20
CRB-701, 2 µM	0.00	0.07	0.14	0.23	0.40	0.74	1.04
**PBST (control)**							
CRB-701, 0.02 µM	0.00	0.00	0.00	0.14	0.10	0.18	0.20
CRB-701, 0.2 µM	0.00	0.03	0.05	0.08	0.10	0.18	0.24
CRB-701, 2 µM	0.00	0.02	0.05	0.08	0.11	0.18	0.25

MMAE release is determined by LC-MS/MS. The measured MMAE concentration at each time point is shown as a percentage of the theoretically expected molality. Mean values are shown for two replicates.

LC-MS/MS, liquid chromatography–tandem mass spectrometry; MMAE, monomethyl auristatin E; PBST, phosphate-buffered saline with 0.1% Tween-20.
