# Supplementary material for: CRB-701: A Second-generation Nectin-4–Targeted Antibody–Drug Conjugate with Optimized Stability and Pharmacokinetics for the Treatment of Solid Tumors
Source: Cancer Res Commun. 2026 Sep 15;6(9):2164–76. doi: 10.1158/2767-9764.CRC-25-0799 (PMC13576400; doi:10.1158/2767-9764.CRC-25-0799)
Supplement: Supplementary file 24 [file crc-25-0799_table_s9_suppst9.pdf]

Table S9. Half-life of CRB-701 (0.02, 0.2, or 2 μM) following incubation in human, cynomolgus monkey, or Sprague Dawley rat plasma

**$T_{1/2}$ (hours)**	**CRB-701, 0.02 μM**	**CRB-701, 0.2 μM**	**CRB-701, 2 μM**
Human	1155	1155	770
Cynomolgus monkey	990	770	866
Sprague Dawley rat	866	770	990

$T_{1/2}$, half-life.
